# Supplementary material for: Non-anaesthetist-administered ketamine for emergency caesarean section in Kenya: cost-effectiveness analysis
Source: BMJ Open. 2022 Oct 5;12(10):e051055. doi: 10.1136/bmjopen-2021-051055 (PMC9535153; doi:10.1136/bmjopen-2021-051055)
Supplement: Supplementary data [file bmjopen-2021-051055supp001.pdf]

Supplementary Material

Table S1. MANDATE Input Parameters and Resulting Caesarean Procedures

OBSTRUCTED LABOR

FIRST Stage OL Treatment	Baseline	Improved	Difference
Penetration rate in the Hospital	*0.75*	*0.99*	
Utilization rate in the Hospital	*0.6*	*0.99*	
Efficacy (% reduction in condition) in the Hospital	1		
Efficacy (% reduction in Other Fetal Death from OL) in the Hospital	0.95		
Efficacy (% reduction in Ruptured Uterus) in the Hospital	0.95		
Efficacy (% reduction in Birth Asphyxia) in the Hospital	0.85		
Efficacy (% reduction in Maternal Sepsis) in the Hospital	0.85		
Efficacy (% reduction in Neonatal SBI) in the Hospital	0.85		
Efficacy (% reduction in PPH Atonic Uterus) in the Hospital	0.85		
Number CS received	3046	6768	3722

SECOND Stage OL Treatment	Baseline	Improved	Difference
Penetration rate in the Hospital	*0.75*	*0.99*	
Utilization rate in the Hospital	*0.6*	*0.99*	
Efficacy (% reduction in condition) in the Hospital	1		
Efficacy (% reduction in Other Fetal Death from OL) in the Hospital	0.95		
Efficacy (% reduction in Ruptured Uterus) in the Hospital	0.95		
Efficacy (% reduction in Birth Asphyxia) in the Hospital	0.85		
Efficacy (% reduction in Maternal Sepsis) in the Hospital	0.85		
Efficacy (% reduction in Neonatal SBI) in the Hospital	0.85		

Resch SC, *et al. BMJ Open* 2022; 12:e051055. doi: 10.1136/bmjopen-2021-051055

Efficacy (% reduction in PPH Atonic Uterus) in the Hospital	0.85		
Number CS	2543	5652	3109
FIRST Stage Protracted OL Treatment (Late CS - efficacy lower)	Baseline	Improved	Difference
Penetration rate in the Hospital	*0.75*	*0.99*	
Utilization rate in the Hospital	*0.95*	*0.99*	
Efficacy (% reduction in condition) in the Hospital	1		
Efficacy (% reduction in Other Fetal Death from OL) in the Hospital	0.1		
Efficacy (% reduction in Ruptured Uterus) in the Hospital	0.7		
Efficacy (% reduction in Birth Asphyxia) in the Hospital	0.2		
Efficacy (% reduction in Maternal Sepsis) in the Hospital	0.4		
Efficacy (% reduction in Neonatal SBI) in the Hospital	0.2		
Efficacy (% reduction in PPH Atonic Uterus) in the Hospital	0.65		
Number CS	4491	3164	-1327
SECOND Stage Protracted OL Treatment	Baseline	Improved	Difference
Penetration rate in the Hospital	*0.75*	*0.99*	
Utilization rate in the Hospital	*0.95*	*0.99*	
Efficacy (% reduction in condition) in the Hospital	1		
Efficacy (% reduction in Other Fetal Death from OL) in the Hospital	0.1		
Efficacy (% reduction in Ruptured Uterus) in the Hospital	0.7		
Efficacy (% reduction in Birth Asphyxia) in the Hospital	0.2		
Efficacy (% reduction in Maternal Sepsis) in the Hospital	0.4		
Efficacy (% reduction in Neonatal SBI) in the Hospital	0.2		
Efficacy (% reduction in Atonic Uterus) in the Hospital	0.65		
Number CS	3134	2746	-388
	Baseline	Improved	Difference
**TOTAL CS FOR OBSTRUCTED LABOR**	**13214**	**18330**	**5116**

PRE-ECLAMPSIA AND ECLAMPSIA

Early Low Rate C-section Technology Interventions	Baseline	Improved	Difference
Penetration	*0.75*	*0.99*	*0.24*
Utilization	*0.4*	*0.99*	*0.59*
Mild PE Maternal Efficacy	0.95		
Mild PE Fetal Efficacy	0.5		
Severe PE Maternal Efficacy	0.8		
SeverePE Fetal Efficacy	0.3		
Mild PE Maternal 1st Seizure Efficacy	0.95		
Severe PE Maternal 1st Seizure Efficacy	0.8		
CS Numbers	1047	3489	2442

Early High Rate C-section Technology Interventions (i.e. C-section or induction (to prevent eclampsia if mother is diagnosed with severe preeclampsia)	Baseline	Improved	Difference
Penetration	*0.75*	*0.99*	*0.24*
Utilization	*0.8*	*0.99*	*0.19*
Mild PE Maternal Efficacy	0.6		
Mild PE Fetal Efficacy	0.6		
Severe PE Maternal Efficacy	0.8		-0.8
SeverePE Fetal Efficacy	0.3		
Mild PE Maternal 1st Seizure Efficacy	0.6		
Severe PE Maternal 1st Seizure Efficacy	0.8		
CS Numbers	982	1636	654

Standard C-section Technology Interventions (for those DIAGNOSED WITH ECLAMPSIA that did not have early CS and that have intra and ante seizure)	Baseline	Improved	Difference
Penetration	*0.75*	*0.99*	*0.24*
Utilization	*0.8*	*0.99*	*0.19*
Maternal 1st&Multiple Seizure Efficacy	0.75		
Mild PE Fetal Efficacy	0.4		
Severe PE Maternal 1st&Multiple Seizure Efficacy	0.75		
Severe PE Fetal Efficacy	0.4		
CS Numbers	189	306	117

Standard C-section Technology Interventions (for those DIAGNOSED WITH ECLAMPSIA that did not have early CS and that have intra and ante seizure)	Baseline	Improved	Difference

Penetration	*0.75*	*0.99*	*0.24*
Utilization	*0.8*	*0.99*	*0.19*
Maternal 1st&Multiple Seizure Efficacy	0.75		
Mild PE Fetal Efficacy	0.4		
Severe PE Maternal 1st&Multiple Seizure Efficacy	0.75		
Severe PE Fetal Efficacy	0.4		
CS Numbers	23	38	15
	Baseline	Improved	Difference
**TOTAL C/S FOR PRE-ECLAMPSIA AND ECLAMPSIA**	**2241**	**5469**	**3228**

ABRUPTION, PREVIA, RUPTURED UTERUS

AIPH due to Significant Abruption Treatment (Early Stage)	Baseline	Improved	Difference
Penetration rate in the Hospital	*0.75*	*0.99*	*0.24*
Utilization rate in the Hospital	*0.2*	*0.99*	*0.79*
Efficacy (% reduction in condition) in the Hospital	0.9		
Efficacy (% reduction in Birth Asphyxia)	0.85		
Efficacy (% reduction in Fetal Deaths)	0.85		
Number CS received	71	475	404

AIPH due to Significant Abruption Treatment (Late Stage)	Baseline	Improved	Difference
Penetration rate in the Hospital	*0.75*	*0.99*	*0.24*
Utilization rate in the Hospital	*0.6*	*0.99*	*0.39*
Efficacy (% reduction in condition) in the Hospital	0.7		
Efficacy (% reduction in Birth Asphyxia)	0.2		
Efficacy (% reduction in Fetal Deaths)	0.1		
Number CS received	424	805	381

Placenta Previa Treatment (Early Stage)	Baseline	Improved	Difference
Penetration rate in the Hospital	*0.75*	*0.99*	*0.24*
Utilization rate in the Hospital	*0.2*	*0.99*	*0.79*
Efficacy (% reduction in condition) in the Hospital	0.9		
Efficacy (% reduction in Birth Asphyxia)	0.85		
Efficacy (% reduction in Fetal Deaths)	0.95		
Number CS received	24	159	135

Placenta Previa Treatment (Late Stage)	Baseline	Improved	Difference
Penetration rate in the Hospital	*0.75*	*0.99*	*0.24*
Utilization rate in the Hospital	*0.6*	*0.99*	*0.39*

Efficacy (% reduction in condition) in the Hospital	0.7		
Efficacy (% reduction in Birth Asphyxia)	0.2		
Efficacy (% reduction in Fetal Deaths)	0.1		
Number CS received	142	263	121
Ruptured Uterus Treatment (Early Stage)	Baseline	Improved	Difference
Penetration rate in the Hospital	*0.75*	*0.99*	*0.24*
Utilization rate in the Hospital	*0.25*	*0.99*	*0.74*
Efficacy (% reduction in condition) in the Hospital	0.75		
Efficacy (% reduction in Birth Asphyxia)	0.85		
Efficacy (% reduction in Fetal Deaths)	0.85		
Number CS received	28	148	120
Ruptured Uterus Treatment (Late Stage)	Baseline	Improved	Difference
Penetration rate in the Hospital	*0.75*	*0.99*	*0.24*
Utilization rate in the Hospital	*0.75*	*0.99*	*0.24*
Efficacy (% reduction in condition) in the Hospital	0.55		
Efficacy (% reduction in Birth Asphyxia)	0.1		
Efficacy (% reduction in Fetal Deaths)	0.1		
Number CS received	203	284	81
	Baseline	Improved	Difference
**TOTAL C/S FOR ABRUPTION, PREVIA, RUPTURED UTERUS**	**892**	**2134**	**1242**

FETAL DISTRESS

SFD- Multiple births during First Stage Labor Treatment	Baseline	Improved	Difference
Penetration rate in the Hospital	*0.75*	*0.99*	*0.24*
Utilization rate in the Hospital	*0.4*	*0.99*	*0.59*
Efficacy (% reduction in condition) in the Hospital	0.9		
Efficacy (% reduction in Birth Asphyxia)	0.5		
Number CS received	52	173	121
SFD- Multiple births during Second Stage Labor Final Treatment	Baseline	Improved	Difference
Penetration rate in the Hospital	*0.75*	*0.99*	*0.24*
Utilization rate in the Hospital	*0.4*	*0.99*	*0.59*
Efficacy (% reduction in condition) in the Hospital	0.9		
Efficacy (% reduction in Birth Asphyxia)	0.5		
Number CS received	21	69	48

SFD- IUGR during First Stage Labor Treatment	Baseline	Improved	Difference
Penetration rate in the Hospital	*0.75*	*0.99*	*0.24*
Utilization rate in the Hospital	*0.4*	*0.99*	*0.59*
Efficacy (% reduction in condition) in the Hospital	0.9		
Efficacy (% reduction in Birth Asphyxia)	0.5		
Number CS received	499	1663	1164

SFD- IUGR during Second Stage Labor Final Treatment	Baseline	Improved	Difference
Penetration rate in the Hospital	*0.75*	*0.99*	*0.24*
Utilization rate in the Hospital	*0.4*	*0.99*	*0.59*
Efficacy (% reduction in condition) in the Hospital	0.9		
Efficacy (% reduction in Birth Asphyxia)	0.5		
Number CS received	199	664	465

SFD- Breech during First Stage Labor Treatment	Baseline	Improved	Difference
Penetration rate in the Hospital	*0.75*	*0.99*	*0.24*
Utilization rate in the Hospital	*0.4*	*0.99*	*0.59*
Efficacy (% reduction in condition) in the Hospital	0.9		
Efficacy (% reduction in Birth Asphyxia)	0.5		
Number CS received	52	172	120

SFD- Breech during Second Stage Labor Final Treatment	Baseline	Improved	Difference
Penetration rate in the Hospital	*0.75*	*0.99*	*0.24*
Utilization rate in the Hospital	*0.4*	*0.99*	*0.59*
Efficacy (% reduction in condition) in the Hospital	0.9		
Efficacy (% reduction in Birth Asphyxia)	0.5		
Number CS received	21	69	48

SFD- Cord Accident during First Stage Labor Treatment	Baseline	Improved	Difference
Penetration rate in the Hospital	*0.75*	*0.99*	*0.24*
Utilization rate in the Hospital	*0.4*	*0.99*	*0.59*
Efficacy (% reduction in condition) in the Hospital	0.9		
Efficacy (% reduction in Birth Asphyxia)	0.5		
Number CS received	203	678	475

SFD- Cord Accident during Second Stage Labor Final Treatment	Baseline	Improved	Difference

Penetration rate in the Hospital	*0.75*	*0.99*	*0.24*
Utilization rate in the Hospital	*0.4*	*0.99*	*0.59*
Efficacy (% reduction in condition) in the Hospital	0.9		
Efficacy (% reduction in Birth Asphyxia)	0.5		
Number CS received	81	271	190

SFD- Other during First Stage Labor Treatment	Baseline	Improved	Difference
Penetration rate in the Hospital	*0.75*	*0.99*	*0.24*
Utilization rate in the Hospital	*0.4*	*0.99*	*0.59*
Efficacy (% reduction in condition) in the Hospital	0.9		
Efficacy (% reduction in Birth Asphyxia)	0.5		
Number CS received	598	1993	1395

SFD- Other during Second Stage Labor Final Treatment	Baseline	Improved	Difference
Penetration rate in the Hospital	0.75	0.99	*0.24*
Utilization rate in the Hospital	0.4	0.99	*0.59*
Efficacy (% reduction in condition) in the Hospital	0.9		
Efficacy (% reduction in Birth Asphyxia)	0.5		
Number CS received	239	796	557
	Baseline	Improved	Difference
**TOTAL C/S FOR FETAL DISTRESS**	**1965**	**6548**	**4583**
**Total Additional CS All Indications Combined**	**18312**	**32481**	**14169**

Table S2. CONSOLIDATED HEALTH ECONOMIC EVALUATION REPORTING STANDARDS (CHEERS) CHECKLIST

CHEERS checklist—Items to include when reporting economic evaluations of health interventions

Section/item	Item No	Recommendation	Reported on Section / Paragraph
**Title and abstract**			
Title	1	Identify the study as an economic evaluation or use more specific terms such as “cost-effectiveness analysis”, and describe the interventions compared.	Title Page
Abstract	2	Provide a structured summary of objectives, perspective, setting, methods (including study design and inputs), results (including base case and uncertainty analyses), and conclusions.	Abstract
**Introduction**			
Background and objectives	3	Provide an explicit statement of the broader context for the study.	Introduction, Paragraphs 1-3
		Present the study question and its relevance for health policy or practice decisions.	Introduction, Paragraph 4
**Methods**			
Target population and subgroups	4	Describe characteristics of the base case population and subgroups analysed, including why they were chosen.	Methods, Paragraphs 1,7
Setting and location	5	State relevant aspects of the system(s) in which the decision(s) need(s) to be made.	Methods, Paragraphs 1
Study perspective	6	Describe the perspective of the study and relate this to the costs being evaluated.	Methods, line 108
Comparators	7	Describe the interventions or strategies being compared and state why they were chosen.	Abstract, Introduction line 50, Methods line 78, line 85
Time horizon	8	State the time horizon(s) over which costs and consequences are being evaluated and say why appropriate.	Methods, line 129
Discount rate	9	Report the choice of discount rate(s) used for costs and outcomes and say why appropriate.	Methods, line 131
Choice of health outcomes	10	Describe what outcomes were used as the measure(s) of benefit in the evaluation and their relevance for the type of analysis performed.	Methods, Paragraphs 33-35
Measurement of effectiveness	11a	*Single study-based estimates:* Describe fully the design features of the single	Not applicable

Section/item	Item No	Recommendation	Reported on Section / Paragraph
		effectiveness study and why the single study was a sufficient source of clinical effectiveness data.	
	11b	*Synthesis-based estimates*: Describe fully the methods used for identification of included studies and synthesis of clinical effectiveness data.	Methods, lines 52-106
Measurement and valuation of preference based outcomes	12	If applicable, describe the population and methods used to elicit preferences for outcomes.	Not applicable
Estimating resources and costs	13a	*Single study-based economic evaluation:* Describe approaches used to estimate resource use associated with the alternative interventions. Describe primary or secondary research methods for valuing each resource item in terms of its unit cost. Describe any adjustments made to approximate to opportunity costs.	Not applicable
	13b	*Model-based economic evaluation:* Describe approaches and data sources used to estimate resource use associated with model health states. Describe primary or secondary research methods for valuing each resource item in terms of its unit cost. Describe any adjustments made to approximate to opportunity costs.	Methods, line 108-127
Currency, price date, and conversion	14	Report the dates of the estimated resource quantities and unit costs. Describe methods for adjusting estimated unit costs to the year of reported costs if necessary. Describe methods for converting costs into a common currency base and the exchange rate.	Methods, line 123-125
Choice of model	15	Describe and give reasons for the specific type of decision-analytical model used. Providing a figure to show model structure is strongly recommended.	Methods, line 52-54, 80-85; Figure 1
Assumptions	16	Describe all structural or other assumptions underpinning the decision-analytical model.	Methods, line 52-166; Figure 1; Tables 1; Suppl material
Analytical methods	17	Describe all analytical methods supporting the evaluation. This could include methods for dealing with skewed, missing, or censored data; extrapolation methods; methods for pooling data; approaches to	Methods, line 52-166, Figure1;

Resch SC, *et al. BMJ Open* 2022; 12:e051055. doi: 10.1136/bmjopen-2021-051055

Section/item	Item No	Recommendation	Reported on Section / Paragraph
		validate or make adjustments (such as half cycle corrections) to a model; and methods for handling population heterogeneity and uncertainty.	
**Results**			
Study parameters	18	Report the values, ranges, references, and, if used, probability distributions for all parameters. Report reasons or sources for distributions used to represent uncertainty where appropriate. Providing a table to show the input values is strongly recommended.	Methods, line 52-166; Tables 1; Figure 1; Table S1, S2, S4, S6 in Supplementary Material
Incremental costs and outcomes	19	For each intervention, report mean values for the main categories of estimated costs and outcomes of interest, as well as mean differences between the comparator groups. If applicable, report incremental cost-effectiveness ratios.	Results, 168-222; Table 4, Table S5
Characterising uncertainty	20a	*Single study-based economic evaluation:* Describe the effects of sampling uncertainty for the estimated incremental cost and incremental effectiveness parameters, together with the impact of methodological assumptions (such as discount rate, study perspective).	Not applicable
	20b	*Model-based economic evaluation:* Describe the effects on the results of uncertainty for all input parameters, and uncertainty related to the structure of the model and assumptions.	Results, line 215-222; Table 4
Characterising heterogeneity	21	If applicable, report differences in costs, outcomes, or cost-effectiveness that can be explained by variations between subgroups of patients with different baseline characteristics or other observed variability in effects that are not reducible by more information.	Not applicable
**Discussion**			
Study findings, limitations, generalisability, and current knowledge	22	Summarise key study findings and describe how they support the conclusions reached. Discuss limitations and the generalisability of the findings and how the findings fit with current knowledge.	Discussion
**Other**			
Source of funding	23	Describe how the study was funded and the role of the funder in the identification, design, conduct, and reporting of the	Funding information page 17

Section/item	Item No	Recommendation	Reported on Section / Paragraph
		analysis. Describe other non-monetary sources of support.	
Conflicts of interest	24	Describe any potential for conflict of interest of study contributors in accordance with journal policy. In the absence of a journal policy, we recommend authors comply with International Committee of Medical Journal Editors recommendations.	Competing interests page 18

Table adapted from the Consolidated Health Economic Evaluation Reporting Standards (CHEERS) Checklist of items to include when reporting economic evaluations of health interventions.[1]

REFERENCE:

1. Husereau D, Drummond M, Petrou S, Carswell C, Moher D, Greenberg D, et al. Consolidated Health Economic Evaluation Reporting Standards (CHEERS)—Explanation and Elaboration: A Report of the ISPOR Health Economic Evaluation Publication Guidelines Good Reporting Practices Task Force. Value Health. 2013;16: 231–250. doi:10.1016/j.jval.2013.02.002

TABLE S3· COUNTY INDICATORS, 2018

Region	County	Target	Skilled birth attendant present (%)	Population CS rate (%)	Facility CS rate (%)	Operating Rooms	Expected Births	Expected Emergency Caesarean delivery	Current Caesarean delivery
Central	Nyandarua	Yes	85·3	9·0	10·6	8	23,830	3,575	2,145
	Murang'a	Yes	85·5	11·7	13·7	7	29,596	4,439	3,463
	Nyeri		88·1	13·5	15·3	12	23,926	3,589	3,230
	Kirinyaga		92·3	15·3	16·6	2	18,930	2,839	2,896
	Kiambu		92·6	20·1	21·7	14	76,295	11,444	15,335
Coast	Tana River	Yes	32·2	2·4	7·5	2	16,816	2,522	404
	Kilifi	Yes	52·3	4·4	8·4	6	71,010	10,652	3,124
	Kwale	Yes	50·1	5·3	10·6	3	41,030	6,155	2,175
	Taita Taveta	Yes	62·5	8·7	13·9	1	10,858	1,629	945
	Lamu		47·3	7·3	15·4	3	5,381	807	393
	Mombasa		82·8	13·3	16·1	18	49,198	7,380	6,543
Eastern	Marsabit	Yes	25·8	2·3	8·9	2	8,744	1,312	201
	Kitui	Yes	46·2	5·4	11·7	5	42,087	6,313	2,273
	Isiolo		43·8	6·9	15·8	2	8,168	1,225	564
	Machakos		63·4	11·4	18·0	7	47,372	7,106	5,400
	Makueni		54·6	9·9	18·1	4	34,112	5,117	3,377
	Tharaka-Nithi		76·6	14·5	18·9	4	13,549	2,032	1,965
	Embu		81·5	15·9	19·5	6	19,314	2,897	3,071
	Meru		82·8	18·5	22·3	9	49,678	7,452	9,190
N· Eastern	Mandera	Yes	38·7	1·8	4·7	3	14,894	2,234	268
	Wajir	Yes	21·7	1·3	6·0	4	24,791	3,719	322

Resch SC, *et al. BMJ Open* 2022; 12:e051055. doi: 10.1136/bmjopen-2021-051055

	Garissa	Yes	39·8	5·5	13·8	21	22,773	3,416	1,253
Nyanza	Homa Bay	Yes	60·4	3·1	5·1	5	63,227	9,484	1,960
	Siaya	Yes	70·4	4·3	6·1	4	37,571	5,636	1,616
	Nyamira	Yes	74·1	5·2	7·0	1	18,737	2,811	974
	Kisii	Yes	72·8	5·3	7·3	13	46,315	6,947	2,455
	Kisumu	Yes	69·2	6·8	9·8	14	48,045	7,207	3,267
	Migori	Yes	53·4	6·5	12·2	5	54,290	8,144	3,529
Rift Valley	Turkana	Yes	22·8	0·5	2·2	4	33,343	5,001	167
	Elgeyo Marakwet	Yes	65·0	3·5	5·4	6	16,143	2,421	565
	Nandi	Yes	46·8	2·8	6·0	4	38,628	5,794	1,082
	Uasin Gishu	Yes	59·0	4·8	8·1	10	46,411	6,962	2,228
	West Pokot	Yes	27·0	2·2	8·1	2	29,019	4,353	638
	Bomet	Yes	52·2	4·8	9·2	8	48,237	7,236	2,315
	Baringo	Yes	53·8	5·2	9·7	4	22,869	3,430	1,189
	Nakuru	Yes	69·5	6·9	9·9	11	87,345	13,102	6,027
	Samburu	Yes	29·0	2·9	10·0	3	11,242	1,686	326
	Narok	Yes	40·3	5·1	12·7	5	61,305	9,196	3,127
	Trans-Nzoia	Yes	41·8	5·3	12·7	3	50,735	7,610	2,689
	Laikipia	Yes	49·5	6·5	13·1	3	20,083	3,012	1,305
	Kericho		64·4	10·3	16·0	9	35,841	5,376	3,692
	Kajiado		63·2	11·1	17·6	8	44,297	6,645	4,917
Western	Kakamega	Yes	48·6	3·1	6·4	6	71,779	10,767	2,225
	Busia	Yes	58·5	4·0	6·8	7	39,301	5,895	1,572
	Bungoma	Yes	41·4	5·0	12·1	7	83,598	12,540	4,180
	Vihiga	Yes	50·3	6·6	13·1	4	22,004	3,301	1,452
Nairobi	Nairobi		89·1	20·7	23·2	51	197,079	29,562	40,795
**KENYA**		**57**	**61·8**	**8·7**	**14·0**	**340**	**1,879,796**	**281,969**	**162,828**
Target Counties		*33*	*52·9*	*4·9*	*9·3*	*191*	*1,256,657*	*188,498*	*61,459*

CS = caesarean delivery procedure

Resch SC, *et al. BMJ Open* 2022; 12:e051055. doi: 10.1136/bmjopen-2021-051055

TABLE S4· ESM-KETAMINE PROGRAM SCALE UP TARGETS

**Year**	**1**	**2**	**3**	**4**	**5**
ESMK providers trained initially	72	144	144	144	69
ESMK provider trained to offset turnover	5	15	25	35	40
Total ESMK providers in service	72	216	360	504	**573**
Absolute number of QA/QC staff	1	2	2	3	4
Facilities "on-line"	24	72	120	168	**191**
Coverage	13%	38%	63%	88%	100%

ESMK= Every Second Matters -Ketamine program, QA/QC = Quality Assurance/ Quality Control

TABLE S5· ESM-KETAMINE PROGRAM COST PROJECTION· BASE CASE, UNDISCOUNTED

Year	1	2	3	4	5	Total
Training (initial)	$61,200	$122,400	$122,400	$122,400	$58,650	$487,050
Training (replacement)	$4,250	$12,750	$21,250	$29,750	$34,000	$102,000
QA/QC	$16,400	$32,800	$32,800	$49,200	$65,600	$196,800
Coordination	$48,000	$48,000	$48,000	$48,000	$48,000	$240,000
Overhead	$19,478	$32,393	$33,668	$37,403	$30,938	$153,880
Total	$149,328	$248,343	$258,118	$286,753	$237,188	**$1,179,730**

QA/QC = Quality Assurance/Quality Control

Fig S1. Annual Cost of Scaling up ESM-Ketamine Program, by Activity

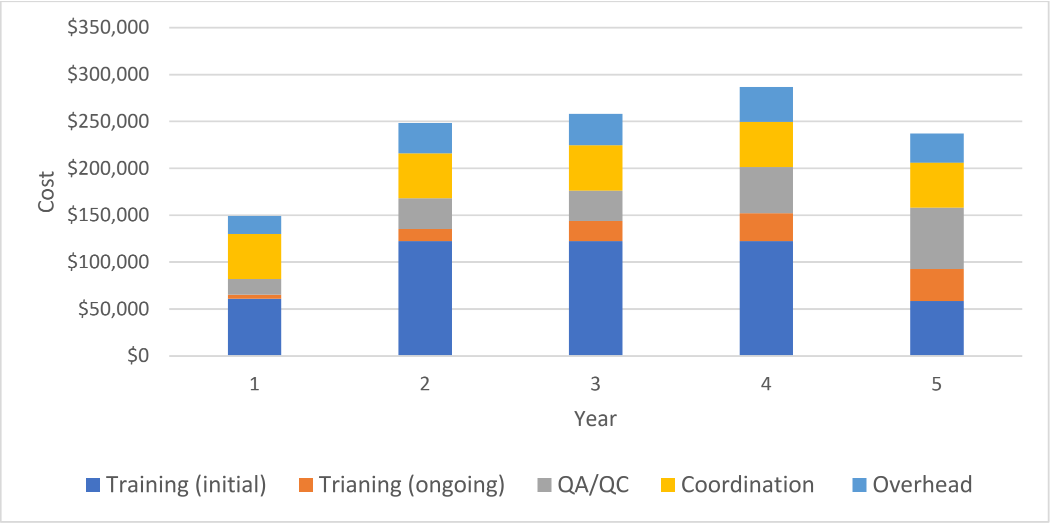

QA/QC = Quality Assurance/Quality Control

Resch SC, *et al. BMJ Open* 2022; 12:e051055. doi: 10.1136/bmjopen-2021-051055

TABLE S6. EXPECTED HEALTH IMPACT OF EMERGENCY CAESAREAN DELIVERY, WEIGHTED AVERAGE LIVES SAVED ACROSS ALL INDICATIONS.

	**Lives Saved**	**Lives saved per Caesarean delivery**	**Number of procedures to save 1 life**
Maternal	154	0·011	92
Foetal	2,339	0·165	6·1
Total	2,493	0·178	5·7

TABLE S7. FOETAL AND MATERNAL DEATHS IN 2017 ATTRIBUTABLE TO COMPLICATIONS THAT INDICATE EMERGENCY CAESAREAN DELIVERY UNDER A BASELINE SCENARIO AND AN "IMPROVED" SCENARIO IN WHICH THERE ARE NO BOTTLENECKS TO EMERGENCY CAESAREAN DELIVERY IN HOSPITAL FACILITIES.

Complication	Foetal Deaths: Baseline	Foetal Deaths: Improved	Foetal Deaths Prevented	Percent of Foetal Deaths Prevented
Foetal Abruption	5,093	4982	111	5%
Foetal Previa	508	494	14	1%
Foetal Ruptured Uterus	1,618	1554	64	3%
Foetal PE/E	6,077	5712	365	16%
SFD MB	488	461	28	1%
SFD IUGR	4,899	4635	264	11%
SFD Breech	498	470	27	1%
SFD Cord	1,996	1889	108	5%
SFD De Novo	5,891	5575	316	14%
Foetal OL	7,195	6665	530	23%
NN Sepsis Term	6,490	6395	95	4%
Term BA	12,318	11900	418	18%
Total Foetal Deaths	53,071	50732	2,339	100%

Complication	Maternal Deaths: Baseline	Maternal Deaths: Improved	Maternal Deaths Prevented	Percent of Maternal Deaths Prevented
Maternal Sepsis	731	717	14	9%
Abruption	348	337	12	8%
Previa	104	100	4	3%
Ruptured Uterus	216	205	11	7%
Atonic Uterus	2,262	2,254	8	5%
PE/E	931	825	106	69%
Total Maternal Deaths	4,592	4,439	154	100%
**Total Deaths**	**57,663**	**55,171**	**2,493**	
