# Supplementary material for: Non-anaesthetist-administered ketamine for emergency caesarean section in Kenya: cost-effectiveness analysis
Source: BMJ Open. 2022 Oct 5;12(10):e051055. doi: 10.1136/bmjopen-2021-051055 (PMC9535153; doi:10.1136/bmjopen-2021-051055)
Supplement: Author's manuscript [file bmjopen-2021-051055.draft_revisions.pdf]

BMJ Open

BMJ Open is committed to open peer review. As part of this commitment we make the peer review history of every article we publish publicly available.

When an article is published we post the peer reviewers' comments and the authors' responses online. We also post the versions of the paper that were used during peer review. These are the versions that the peer review comments apply to.

The versions of the paper that follow are the versions that were submitted during the peer review process. They are not the versions of record or the final published versions. They should not be cited or distributed as the published version of this manuscript.

BMJ Open is an open access journal and the full, final, typeset and author-corrected version of record of the manuscript is available on our site with no access controls, subscription charges or pay-per-view fees (http://bmjopen.bmj.com).

If you have any questions on BMJ Open's open peer review process please email info.bmjopen@bmj.com

BMJ Open

Cost-effectiveness of Ketamine Anaesthesia for Emergency Caesarean Section in Kenya

Journal:	*BMJ Open*
Manuscript ID	bmjopen-2021-051055
Article Type:	Original research
Date Submitted by the Author:	06-Jan-2022
Complete List of Authors:	Resch, Stephen; Harvard University T H Chan School of Public Health, Center for Health Decision Science<br>Suarez, Sebastian ; Massachusetts General Hospital; Boston University<br>Omotayo, Moshood Olanrewaju ; Massachusetts General Hospital; Harvard Medical School<br>Griffin, Jennifer; RTI International, Center for Global Health<br>Sessler, Daniel; Cleveland Clinic, Department of Outcomes Research<br>Burke, Thomas; Harvard Medical School, Division of Global Health and Human Rights, Department of Emergency Medicine, Massachusetts General Hospital; Harvard University T H Chan School of Public Health
Keywords:	Health economics < HEALTH SERVICES ADMINISTRATION & MANAGEMENT, Anaesthesia in obstetrics < ANAESTHETICS, PUBLIC HEALTH

SCHOLARONE™
Manuscripts

I, the Submitting Author has the right to grant and does grant on behalf of all authors of the Work (as defined in the below author licence), an exclusive licence and/or a non-exclusive licence for contributions from authors who are: i) UK Crown employees; ii) where BMJ has agreed a CC-BY licence shall apply, and/or iii) in accordance with the terms applicable for US Federal Government officers or employees acting as part of their official duties; on a worldwide, perpetual, irrevocable, royalty-free basis to BMJ Publishing Group Ltd ("BMJ") its licensees and where the relevant Journal is co-owned by BMJ to the co-owners of the Journal, to publish the Work in this journal and any other BMJ products and to exploit all rights, as set out in our licence.

The Submitting Author accepts and understands that any supply made under these terms is made by BMJ to the Submitting Author unless you are acting as an employee on behalf of your employer or a postgraduate student of an affiliated institution which is paying any applicable article publishing charge ("APC") for Open Access articles. Where the Submitting Author wishes to make the Work available on an Open Access basis (and intends to pay the relevant APC), the terms of reuse of such Open Access shall be governed by a Creative Commons licence – details of these licences and which Creative Commons licence will apply to this Work are set out in our licence referred to above.

Other than as permitted in any relevant BMJ Author's Self Archiving Policies, I confirm this Work has not been accepted for publication elsewhere, is not being considered for publication elsewhere and does not duplicate material already published. I confirm all authors consent to publication of this Work and authorise the granting of this licence.

For peer review only

Cost-effectiveness of Ketamine Anaesthesia for Emergency Caesarean Section in Kenya

Stephen C. Resch, Ph.D., M.P.H. Harvard TH Chan School of Public Health, Boston, MA

Sebastian Suarez, M.D. M.P.H. Massachusetts General Hospital and Boston University, Boston, MA

Moshood Olanrewaju Omotayo, Ph.D. Massachusetts General Hospital and Harvard Medical School, Boston, MA

Jennifer B. Griffin, Ph.D., MPH Center for Global Health, RTI International, Research Triangle Park, NC

Daniel I. Sessler, M.D., Department of Outcomes Research, Cleveland Clinic, Cleveland, OH

Thomas F. Burke, M.D., Department of Emergency Medicine, Massachusetts General Hospital; Harvard Medical School; and Harvard TH Chan School of Public Health, Boston, MA

Corresponding Author: Stephen C. Resch, sresch@hsph.harvard.edu, +1(617) 432-1566

Center for Health Decision Science, Harvard T.H. Chan School of Public Health, 718 Huntington Avenue, Boston MA 02115

Article Type: Original Research

Word Count: Abstract – 291, Main Text -- 3079

Synopsis:

Scaling up a program to train clinicians to administer ketamine as anaesthesia is a cost-effective approach to enabling life-saving emergency caesarean deliveries in Kenya.

For peer review only

Abstract

Objectives: Lack of anaesthesia services is a frequent barrier to emergency surgeries such as caesarean delivery in Kenya. This study aimed to estimate the health impact and cost-effectiveness of scaling up the Every Second Matters (ESM) – Ketamine program that trains non-anaesthetist providers to administer and monitor ketamine during emergency caesarean deliveries.

Setting: Hospitals in Kenyan counties with low rates of caesarean delivery.

Participants: Patients needing emergency caesarean delivery in settings without availability of standard anaesthesia service.

Interventions: Scale up of the ESM-Ketamine program over five years (2020-24) was compared to status quo.

Outcome measures: Cost of implementing the program and corresponding additional emergency caesarean deliveries. Maternal and foetal/neonatal deaths prevented, and corresponding life-years gained due to increased provision of emergency caesarean procedures. Cost-effectiveness was assessed by comparing the cost per life-year gained of the ESM-Ketamine program compared to status quo.

Results: Over five years, the expected gap in emergency caesarean deliveries was 157,000. A $1.2 million ESM-Ketamine program reduced this gap by 28,700, averting by 316 maternal and 4736 foetal deaths and generating 331,000 total life-years gained. Cost-effectiveness of scaling up the ESM-Ketamine program was $44 per life year gained in the base case and $251 in the most pessimistic scenario—a very good value for Kenya at less than 20% of per capita GDP per life-year gained.

Conclusion: In areas of Kenya with significant under-provision of emergency caesarean delivery due to a lack of availability of traditional anaesthesia, an ESM-Ketamine program is likely to enable a substantial number of life-saving surgeries at reasonable cost.

For peer review only

Strengths and limitations of this study

- This study uses a decision analytic approach to synthesize data on costs and outcomes of a long-running ESM-Ketamine program implemented in one county in Kenya and simulate the impact and cost-effectiveness of expanding the program throughout the country.
- The increased provision of emergency caesarean delivery enabled by the ESM-Ketamine program was translated to maternal and foetal deaths averted and life-year gains using the MANDATE model to simulate outcomes of deliveries with complications when surgery is and is not available.
- A limitation of this study is the uncertainty about the extent to which lack of available anaesthesia is the key barrier to timely provision of emergency surgeries in Kenyan health facilities.
- Another limitation of this study is uncertainty about the pace at which the supply of conventional anaesthesiology services can be expanded, such that the ESM-Ketamine program would no longer be needed.

For peer review only

Introduction

The World Health Organization (WHO) estimates that at least 10-15% and possibly as many as 19%(1,2) of all childbirths require caesarean deliveries, most on an emergency basis. Few developing countries provide this level of caesarean deliveries, with considerable consequent morbidity and mortality. Kenya provides more caesarean deliveries than most sub-Saharan African countries, with a population rate of 8.7% (3,4). However, this average rate masks substantial heterogeneity. In 16 of 47 counties the population caesarean delivery rate is below 5%. In 26 counties the rate is between 5% and 15%. In just five counties, mostly those with large urban areas such as Nairobi, the population caesarean delivery rate is over 15%. While rigorous data is lacking, a substantial portion of caesarean procedures contributing to the rate observed in urban areas may be elective (5).

Undersupply of emergency caesarean deliveries in many parts of Kenya likely contributes to the country's high maternal and perinatal mortality. When last measured in 2014, the maternal mortality ratio (MMR) in Kenya was 362 (95% CI: 254, 471) per 100,000 births (4). Perinatal mortality, defined as pregnancy losses occurring after seven completed months of gestation (stillbirths) plus deaths to live births within the first seven days of life (early neonatal deaths), was 29 per 1000 births in 2014 (4,6).

One of the major limitations to provision of caesarean deliveries is lack of anaesthesia. The Lancet Commission on Global Surgery indicated substantially improved health outcomes associated with increases in specialist surgical workforce (surgeons, anaesthetists, and obstetricians) up to at least 20 per 100,000 population, and the

For peer review only - http://bmjopen.bmj.com/site/about/guidelines.xhtml

World Federation of Societies of Anaesthesiologists has indicated about half of these providers – i.e. 10 per 100,000 population—should be anaesthesia providers (7,8). In Kenya, there are less than 2 anaesthesia providers per 100,000 population, of which about 80% are non-physician anaesthetists including clinical officers and nurses (7). The lack of anaesthesia services for emergency surgery is especially grave when considering that the workforce is maldistributed within the country in proportion to the population (9).

We have previously described the Every Second Matters for Emergency and Essential Surgery – Ketamine (ESM-Ketamine) program which has been operating in rural Kenya since 2013 (10,11). While not envisioned as a long-term solution to the shortage of anaesthesia services in Kenya, or a substitute for other health system strengthening efforts aimed at increasing the supply and improving the distribution of anaesthetists and anaesthesiologists, the ESM-Ketamine program is a relatively inexpensive and rapid approach to ensure anaesthesia is available for surgery in emergency situations. Through this program, non-anaesthetist providers are trained to administer and monitor ketamine during emergency surgeries such as caesarean delivery when no anaesthetist is available, enabling procedures that would have not been possible, would have been significantly delayed, or would have been performed without any anaesthesia. The program includes an intensive hands-on five-day training, ESM-Ketamine kits, wallcharts, checklists, and regular supervisory visits for quality assurance. The ESM-Ketamine pilot program in Kenya has proven remarkably safe, with positive patient experiences, no deaths or major adverse events attributed to ketamine in more than 2,000 emergency and essential surgeries including about 450 caesarean deliveries

For peer review only

across 17 hospitals (12,13). However, there are distinct training, implementation, and running costs associated with the ESM-Ketamine program. In this analysis, we model the potential health impact and cost-effectiveness of scaling up the ESM-Ketamine program for emergency caesarean sections throughout Kenya.

Methods

For peer review only

Using data from the 2014 Kenya Demographic and Health Survey (4), we estimated the facility caesarean delivery rate (caesarean delivery performed divided by deliveries with a skilled provider) for all counties in Kenya and identified those where the facility caesarean delivery rate was less than 15%. For these counties, we estimated the number of facility-based deliveries over a five-year period (2020-2024) using UN projections of annual births and calculated the county-specific gap in emergency caesarean delivery using an expected unconstrained emergency caesarean delivery rate of 15% as the reference level.

Under-provision of caesarean delivery can have several causes including lack of functional operating theatre infrastructure, lack of doctors able to perform caesarean procedures, and lack of available anaesthesia service. Ketamine-based anaesthesia via providers trained in the ESM-Ketamine program addresses only one of several possible bottlenecks to emergency caesarean delivery. Thus, the impact of the program will depend on the frequency with which the availability of traditional anaesthesia is the sole bottleneck. While no studies have measured this precisely at a patient case level, there is evidence that in some settings in Kenya, anaesthesia service is in shorter supply than other necessary service components for emergency caesarean. A 2011 study in

For peer review only - http://bmjopen.bmj.com/site/about/guidelines.xhtml

Nyanza region found that for 34 operating theatres with associated doctors able to perform caesarean delivery, there were only 18 anaesthetists and 44% of theatres reported severely limited access to anaesthetist services(14). More recently, in 2014, we surveyed all 30 operating theatres in 2 counties of Western Kenya and found 57% had no access to anaesthesia services (15). Based on this evidence, we assumed that anaesthesia availability was the sole bottleneck in 30% of the cases in which an emergency caesarean delivery could not be provided.

We modelled a gradual five-year scale up of the ESM-Ketamine program that reaches full coverage of all facilities with operating rooms in the target counties (Table 1). Based on the level of scale to be achieved each year, the number of additional emergency caesarean deliveries was calculated for each of the five years.

We used the MANDATE model (www.mandate4mnh.org) to estimate the expected number of lives saved per additional emergency caesarean delivery in Kenya. This model synthesizes evidence on the incidence, case fatality rate, and efficacy of caesarean delivery for all major delivery complications for which emergency caesarean delivery is the recommended intervention(16,17). We simulated two scenarios with this model: a base case approximating status quo for Kenya in 2017 and an alternative case where caesarean delivery is available (and used when indicated) in 99% of emergency cases occurring in or transferred to hospital settings. We calculated the difference in caesarean delivery procedures performed and the difference in foetal and maternal deaths, and then calculated a ratio of deaths prevented per additional caesarean delivery procedure. We applied these ratios to the number of additional caesarean

For peer review only - http://bmjopen.bmj.com/site/about/guidelines.xhtml

deliveries we estimated would occur in Kenya each year as a result of the scale-up of the ESM-Ketamine program.

We converted deaths averted to years of life gained using the most recently available 2016 Kenya life tables. For foetal deaths prevented, we used the average of male and female life expectancy at less than one year of age. For maternal deaths prevented, we used the average of remaining life expectancy for three female age groups (20-24, 25-29, 30-34 years).

Evaluation of the ESM-Ketamine program in 2059 Kenyan patients identified only occasional mild adverse events associated with Ketamine such as hallucinations and salivation, and no major adverse events.[7] We therefore assumed that ESM-Ketamine does not cause major morbidities and did not include the minor consequences of the occasional mild and time-limited adverse events since they pale in comparison to the consequences of not providing emergency caesarean delivery.

Costs

Based on the experience of the current ESM-Ketamine program in Kenya, we modelled the cost of scaling up access. We assumed three ESM-Ketamine trained providers would be required in each facility with surgical capacity to ensure that availability of anaesthesia services would not be a bottleneck to emergency caesarean delivery. In the first year, 72 providers are expected to graduate from the training program. In subsequent years, a second training centre is added, and the number of providers trained per year increases to 144. In the fifth year, the number of newly trained reduces as the necessary supply of ESM-Ketamine providers is reached. We assumed a 7%

For peer review only - http://bmjopen.bmj.com/site/about/guidelines.xhtml

annual staff turnover rate and included additional training to maintain target supply of active ESM-Ketamine providers. The unit cost of training one provider was $650 plus $150 for travel and lodging. In addition, each facility requires a $150 ESM-Ketamine kit (Table 1 and S2). We included cost for supervision and coordination, quality assurance (QA) activities, and overhead. These costs included a full-time program director, one quality assurance (QA) officer per 60 facilities, and 15% overhead.

The cost of additional caesarean delivery procedures ($367 per delivery) was based on a recent rigorous cost analysis conducted in Rwanda adjusted for Kenya based on differences in purchasing power parity (18). Because some of cost would be incurred even if no caesarean procedure was performed, we subtracted the cost of a vaginal delivery – which we assumed was half the cost of a caesarean delivery (19).

Cost-effectiveness Analysis

To calculate the incremental cost per life year gained, we divided the five-year program cost (in 2017 US dollars) by the number of life years gained due to lives saved in the five-year period, discounting both costs and health outcomes by 3% per year. We also reported undiscounted results. Kenya's GDP per capita in 2018 was US$1620 (20). Interventions costing less than 25% of GDP per capita per disability-adjusted life-year (DALY) averted are generally considered highly cost-effective by most standards (21). Our model estimated life-years gained but did not account for disability in those years. Therefore, we used 20% GDP per capita per life year gained ($325 in Kenya) as a benchmark for good value, which is approximately equivalent to assuming an average

For peer review only - http://bmjopen.bmj.com/site/about/guidelines.xhtml

For peer review only

lifetime health-related quality of life of 0.85 (where one represents perfect health and zero represents death) (22).

Patient and Public Involvement

No patient involvement.

Ethical Clearance

This model-based study did not collect any new information from human subjects or use any patient-level data, so we did not obtain approval of an Institutional Review Board. Neither patients nor the public were not involved in the design, conduct, reporting, or dissemination plans of our research.

Sensitivity Analysis

The impact and value of the ESM-Ketamine program will depend on the actual number of emergency caesarean deliveries that are enabled by the program; which in turn depends both on the number of emergency cases requiring caesarean delivery that are currently not receiving caesarean delivery as well as the portion of these cases in which lack of anaesthesia is the sole bottleneck preventing the caesarean delivery procedure from being performed. In sensitivity analysis, we considered scenarios in which only 10% of all hospital deliveries require emergency caesarean delivery (vs. 15% rate assumed in base case), and scenarios in which lack of anaesthesia was the sole bottleneck for only 10% of cases in which emergency caesarean delivery was indicated but not provided (vs. 30% in the base case). We also considered the case in which the

For peer review only

For peer review only - http://bmjopen.bmj.com/site/about/guidelines.xhtml

ESM-Ketamine program costs and the additional delivery costs associated with caesarean section were twice as high as in the base case.

Results

Program scope and cost

In Kenya, 33 counties met the criteria of having a facility caesarean delivery rate less than 15% (Table S1). The emergency caesarean delivery gap across these counties was 157,000 procedures over five years, of which 47,000 are attributable to lack of anaesthesia. The target counties contain 191 hospitals with operating rooms. The modelled ESM-Ketamine program would train 693 providers, enough to maintain 3 providers per facility, while accounting for staff turnover. The scale up of the ESM-Ketamine program from 13% coverage in Year 1 to 100% coverage in Year 5 would enable 28,700 emergency caesarean deliveries, reducing the overall gap in emergency caesarean delivery by 18% and the anaesthesia-attributable gap by 61%. The cost of the five-year scale up of the ESM-Ketamine program is estimated to be about $1.2 million (undiscounted) (Table S3). About half of this cost is the direct cost of training providers (Figure S1).

Health Outcomes

Results from the MANDATE model indicate that the most common indications for emergency caesarean delivery are obstructed labour, pre-eclampsia or eclampsia, and foetal distress (Table 2). In the baseline scenario, the hospital caesarean delivery rate was 7.1%. By maximizing the availability and use of caesarean delivery for emergency

For peer review only - http://bmjopen.bmj.com/site/about/guidelines.xhtml

indications in hospital settings (without changing care-seeking patterns or the effectiveness of the emergency medical transport system) the number of caesarean deliveries almost doubles—to a rate of 12.3% – which corresponds well to the level of emergency caesarean delivery expected based on the incidence of life-saving indications found in an observational study (23).

Analysing the corresponding reduction in maternal and foetal death for improved access to caesarean delivery compared to the status quo, we found that 0.178 deaths would be averted per additional emergency caesarean procedure provided (Table 2 and S4). Over 90% of these deaths were foetal. One foetal death was prevented by every 6.1 emergency caesarean delivery procedures. One maternal death was averted by every 92 emergency caesarean delivery procedures. These estimates represent a weighted average across the emergency indications and accounts for condition-specific mortality risk reduction conferred by caesarean delivery (Table S5).

Applying these rates of life-saving to the additional 28,700 caesarean deliveries provided over five years as a result of the scale-up of the ESM-Ketamine program, we estimated that maternal and foetal deaths would be reduced by 316 and 4736 respectively (Table 3). Considering remaining life expectancies at the moment these deaths are averted, the mortality reductions would translate into 331,000 total life-years gained.

Cost-effectiveness

Cost-effectiveness results are shown in Table 4. Without discounting, the average cost per death prevented by ketamine-enabled caesarean delivery performed during the five-

For peer review only - http://bmjopen.bmj.com/site/about/guidelines.xhtml

year period in the base case was about $1,270. Considering the expected years of life gained by preventing deaths, and discounting both costs and future life-years, the cost-effectiveness of scaling up the ESM-Ketamine program was $44 per life year gained.

In sensitivity analysis, when the caesarean delivery gap among hospital deliveries is based on a bottleneck-free caesarean delivery rate of 10%, and the portion of the gap attributable to lack of anaesthesia is only 10%, an ESM-Ketamine program would prevent 28 maternal and 426 foetal deaths, translating to 29,750 life years gained, and a cost-effectiveness of $125 per life-year gained. In the most pessimistic scenario considered, combining lower impact on caesarean section uptake, higher caesarean procedure cost, and a doubling of the cost of the ESM-Ketamine program itself, the cost-effectiveness ratio would increase to $251 per life year gained.

Discussion

Our analysis shows an ESM-Ketamine program could enable an additional 28,700 emergency caesarean deliveries over five years, reducing the projected gap in emergency caesarean deliveries in 33 Kenyan counties by about 18%. The cost of the ESM-Ketamine program would be about $1700 per ESM-Ketamine provider trained and $41 per emergency caesarean delivery enabled. The total incremental cost, when including the additional cost of the caesarean procedure itself, is about $224 per delivery.

Given that we found one death is expected to be prevented by every six emergency caesarean delivery procedures, the program is likely to be highly cost-effective. In the

For peer review only - http://bmjopen.bmj.com/site/about/guidelines.xhtml

base case, the scale up of ESM-Ketamine program over five years cost $44 per life year gained. In the most pessimistic sensitivity analysis we conducted, the cost increased to $251 per life year gained. These cost-effectiveness ratios are far less than 20% of GDP per capita ($325) benchmark we used as a threshold value and compare favourably to many public health interventions being implemented in Kenya. Indeed, using $325 per life year as a threshold for good value, the ESM-Ketamine program would only have to enable 1720 emergency caesarean deliveries and avert about 300 deaths in five years to be considered cost-effective — which is only about 6% of the impact we estimate.

We did not consider secular changes in the portion of deliveries in health facilities, changes in the availability of timely emergency transport to hospitals with capacity for caesarean delivery, or reduction in the caesarean delivery gap due to increasing availability of traditional anaesthesia services. If more deliveries were to occur in health facilities over time, then there would be more opportunities for the ESM-Ketamine program to enable life-saving emergency caesarean delivery. If the expansion of traditional anaesthesia service reduces the gap in caesarean delivery, the ESM-Ketamine program would be less cost-effective and, ideally, unnecessary. However, the payback period for an ESM-Ketamine program is very short. It is highly unlikely that the investment in establishing a program and training ESM-Ketamine providers would be rendered moot by a sudden expansion of traditional anaesthesia services. For example, in the base case, each ESM-Ketamine trained provider would enable about 41 emergency caesarean deliveries on average over the five-year time horizon, resulting in about 7.3 lives saved per ESM-Ketamine provider trained. Yet, to meet standard benchmarks for cost-effectiveness, each ESM-Ketamine provider would only need to

For peer review only - http://bmjopen.bmj.com/site/about/guidelines.xhtml

enable about three emergency caesarean deliveries — a number that might reasonably be expected within a few months of completing training.

The expected cost of the ESM-Ketamine program is small relative to the cost of caesarean delivery procedures enabled by the availability of ketamine anaesthesia. Therefore, the value of the ESM-Ketamine program is driven in large part by the value of emergency caesarean delivery itself. The overall cost-effectiveness of ESM-Ketamine was much more sensitive to the additional cost of caesarean delivery compared to vaginal delivery than the cost of the ESM-Ketamine program itself.

Although caesarean delivery is also likely to prevent morbidity in both mothers and new-borns (e.g. long-term cognitive problems associated with non-fatal birth asphyxia), we did not include these health benefits, making our analysis conservative.

We did not consider the value of the ESM-Ketamine program related to surgical procedures other than emergency caesarean deliveries which is likely to be substantial. In the current small-scale ESM-Ketamine program in Kenya, emergency caesarean deliveries account for about 20% of the total procedures performed with many of the remaining procedures being for acute abdomens, open fractures, and similarly serious conditions. For all of these reasons, it is likely that we have substantially under-estimated the total value of the ESM-Ketamine program.

Our analysis supports the relatively modest investment needed for scaling up the ESM-Ketamine program over the next five years. Cost per life year gained ranged from $44 (2.8% of GDP per capita) in the base case to $251 (16% of GDP per capita) in our most pessimistic scenario. There remains significant uncertainty about the overall scale of the

For peer review only - http://bmjopen.bmj.com/site/about/guidelines.xhtml

impact of an ESM-Ketamine program due to a lack of data regarding the extent to which anaesthesia is a key bottleneck preventing access to emergency caesarean delivery. Nevertheless, we found that the ESM-Ketamine program would be worthwhile even if the number of emergency caesarean procedures it enabled were an order-of-magnitude smaller than expected in our base case. In areas of Kenya with significant under-provision of emergency caesarean delivery due to a lack of availability of traditional anaesthesia, an ESM-Ketamine program is likely to enable a substantial number of life-saving surgeries at modest cost.

For peer review only

For peer review only - http://bmjopen.bmj.com/site/about/guidelines.xhtml

Author contributions

The study was conceptualized and designed by SR, TB, and SS. SS and MO assisted in gathering information from the ESM-Ketamine pilot project to inform parameters of the cost-effectiveness model. JG conducted analysis with the Mandate model to inform parameters regarding the impact of emergency caesarean section on neonatal and maternal deaths. SR developed the cost-effectiveness model and conducted the analysis. SR, with DS and TB, wrote the manuscript. All authors reviewed and edited manuscript drafts.

Funding information

The work presented in this manuscript was funded by USAID Award No. 7200AA18CA00002 (project title: “Scaling Up Every Second Matters for Mothers and Babies – Ketamine (ESM-Ketamine) in Kenya”) and supported by the Ujenzi Charitable Trust (Grant number: Not Applicable) , Elrha’s Research for Health in Humanitarian Crises (R2HC) Program (Grant number: Not Applicable) and the Saving Lives at Birth partners (Grant number: Not Applicable). The Saving Lives at Birth partners are: United States Agency for International Development (USAID), the Government of Norway, the Bill & Melinda Gates Foundation, Grand Challenges Canada, the UK Government, and the Korea International Cooperation Agency (KOICA). The R2HC program is funded by the UK Foreign, Commonwealth and Development Office (FCDO), Wellcome, and the UK National Institute for Health Research (NIHR). This manuscript was prepared by the listed authors and does not necessarily reflect the views of the funding partners. The

For peer review only - http://bmjopen.bmj.com/site/about/guidelines.xhtml

funding sources had no role in the collection, analysis, or interpretation of the data nor in manuscript preparation.

Competing interests

The authors have no conflicts of interest.

For peer review only

For peer review only - http://bmjopen.bmj.com/site/about/guidelines.xhtml

References

For peer review only

For peer review only - http://bmjopen.bmj.com/site/about/guidelines.xhtml

TABLE 1. KEY MODEL INPUTS

Parameter	Base case Value	Alternative Values	Source Notes
Demographics			
Estimated deliveries (2020)	1.503 million		UN Population Division (derived from crude birth rate and total population estimate)
Change in total deliveries	0.01		Imputed from UN Population Prospects Births (Medium variant)
Target emergency caesarean delivery rate	15%	10%	
Share of emergency caesarean delivery gap attributable only to lack of anaesthesia services	30%	10%	14 , 15
Training Program Implementation Assumptions			
Training centres	1 in first year, 2 thereafter		
Providers trained per session	6		
Trainings per year per training centre	12		
ESMK provider turnover rate	0.07		
ESMK providers per facility	3		
ESM-Ketamine Program Unit Costs			
Training			
Training costs	$650		TTS grant (Jan 2017)
Travel and lodging	$150		MGH / USAID
Kit (1 per facility)	$150		TTS grant
Program Coordination			
Program director	$48,000		TTS grant
QA/QC			

For peer review only - http://bmjopen.bmj.com/site/about/guidelines.xhtml

Facilities per staff person	60		MGH / USAID
QA/QC staff salary	$16,400		TTS grant
Overhead	15%		TTS grant
Life expectancy from age x	Male	Female	WHO Life Tables Kenya 2016
<1 year	64.4	68.9	
20-24 years		53.3	
25-29 years		48.8	
30-34 years		44.4	
Life expectancy per averted death	Undiscounted	Discounted	
Mothers (average of 3 age groups)	48.8	26.2	
Newborns (average of Male and Female <1 year)	66.7	29.5	
Discount Rate, annual	3%		
ESMK = Every Second Matters – Ketamine program; QA/QC = Quality assurance/Quality control; TTS =			

For peer review only

TABLE 2. NUMBER OF EMERGENCY CAESAREAN DELIVERY PROCEDURES BY INDICATION UNDER A BASELINE SCENARIO AND AN "IMPROVED" SCENARIO IN WHICH THERE ARE NO BOTTLENECKS TO EMERGENCY CAESAREAN DELIVERY IN HOSPITAL FACILITIES*

Indication	Baseline	Improved	Difference	Percent of Total Difference
Obstructed Labor	13,214	18,330	5116	36%
Pre-eclampsia / Eclampsia	2241	5469	3228	23%
AIPH - Abruption	495	1280	785	6%
AIPH - Placenta Previa	166	422	256	2%
AIPH - Ruptured Uterus	231	432	201	1%
SFD - MB	73	242	169	1%
SFD - IUGR	698	2327	1629	11%
SFD - Breech	73	241	168	1%
SFD - Cord	284	949	665	5%
SFD - Other	837	2789	1952	14%
Total Procedures	18,312	32,481	14,169	100%
**Deaths Averted**				
Maternal			154	6.2%
Foetal			2339	93.8%
Total			2493	
**Deaths Averted per Emergency Caesarean**				
Maternal			0.011	
Foetal			0.165	
Total			0.178	

*The total number of hospital deliveries was 258,630. In the "baseline" scenario, the emergency caesarean delivery rate is about 7.1% for hospital deliveries, and in the "improved scenario" --representing 99% coverage of emergency caesarean delivery in hospital setting-- the emergency caesarean delivery rate increases to 12.3% of hospital deliveries. In both scenarios, the analysis assumed no non-emergency caesarean deliveries occur for reasons such as maternal request, revenue maximization, or scheduling convenience.

AIPH = Ante/intrapartum Haemorrhage; SFD = Significant foetal distress; MB = multiple births; IUGR = intrauterine growth rate

For peer review only - http://bmjopen.bmj.com/site/about/guidelines.xhtml

TABLE 3. PROGRAMMATIC AND HEALTH OUTCOMES BY YEAR. BASE CASE, UNDISCOUNTED

Year	2020	2021	2022	2023	2024	TOTAL
CS gap	9248	9341	9434	9528	9624	47175
Coverage scale-up	13%	38%	63%	88%	100%	61%
Additional CS provided	1202	3549	5943	8385	9624	28,704
Lives Saved						
Maternal	13	39	65	92	106	316
Foetal	198	586	981	1384	1588	4736
Total	212	625	1046	1476	1694	5052
Life Years Gained						
Maternal	646	1907	3193	4504	5170	15419
New-born	13,222	39,034	65,362	92,212	105,834	315,664
Total	13,867	40,941	68,554	96,716	111,004	331,083
Economic outcomes						
ESMK program	$149,328	$248,343	$258,118	$286,753	$237,188	$1,179,730
Additional CS	$219,791	$648,892	$1,086,553	$1,532,902	$1,759,353	$5,247,491
CS Share of Total Cost	60%	72%	81%	84%	88%	82%
Total Cost	$369,119	$897,235	$1,344,671	$1,819,655	$1,996,541	$6,427,221

CS = emergency caesarean section delivery procedure; ESMK = Every Second Matters – Ketamine program

For peer review only - http://bmjopen.bmj.com/site/about/guidelines.xhtml

TABLE 4. COST-EFFECTIVENESS RESULTS WITH SENSITIVITY ANALYSIS FOR KEY MODEL UNCERTAINTIES

	Base case impact	Base case + higher CS cost	Base case + higher CS cost and ESMK program Cost	Pessimistic impact	Pessimistic impact + higher CS cost	Pessimistic impact + higher CS cost and ESMK program Cost
**Input Parameters**						
Population rate of Emergency CS	15%	15%	15%	10%	10%	10%
Emergency CS gap attributable to anaesthesia	30%	30%	30%	10%	10%	10%
Incremental CS procedure cost*	$183	$366	$366	$183	$366	$366
Program Cost Multiplier	1x	1x	2x	1x	1x	2x
**Outcomes**						
Undiscounted Program Cost	$1.18m	$1.18m	$2.36m	$1.18m	$1.18m	$2.36m
Undiscounted Total Cost	$6.43m	$11.68m	$12.85m	$1.65m	$2.12m	$3.30m
Additional Emergency CS	28,704	28,704	28,704	2,580	2,580	2,580
Maternal Lives Saved	316	316	316	28	28	28
Fetal Lives Saved	4,736	4,736	4,736	426	426	426
**Incremental Cost-effectiveness Ratio**						
Cost per LY (discounted)	$44	$79	$87	$125	$161	$251
GDPpc per LY (discounted)	2.8%	5.1%	5.6%	8.0%	10.3%	16.1%
Cost per Maternal LY (discounted)	$779	$1,412	$1,557	$2,246	$2,880	$4,492
GDPpc per Maternal LY (discounted)	50%	91%	100%	144%	185%	288%

*excess cost of Cesarean delivery cost above the cost of normal vaginal delivery
CS = cesarean delivery procedure, LY = life-year gained, ESMK = Every Second Matters -Ketamine program

For peer review only - http://bmjopen.bmj.com/site/about/guidelines.xhtml

Supplementary Material

TABLE S1· COUNTY INDICATORS, 2018

Region	County	Target	Skilled birth attendant present (%)	Population CS rate (%)	Facility CS rate (%)	Operating Rooms	Expected Births	Expected Emergency Caesarean delivery	Current Caesarean delivery
Central	Nyandarua	Yes	85·3	9·0	10·6	8	23,830	3,575	2,145
	Murang'a	Yes	85·5	11·7	13·7	7	29,596	4,439	3,463
	Nyeri		88·1	13·5	15·3	12	23,926	3,589	3,230
	Kirinyaga		92·3	15·3	16·6	2	18,930	2,839	2,896
	Kiambu		92·6	20·1	21·7	14	76,295	11,444	15,335
Coast	Tana River	Yes	32·2	2·4	7·5	2	16,816	2,522	404
	Kilifi	Yes	52·3	4·4	8·4	6	71,010	10,652	3,124
	Kwale	Yes	50·1	5·3	10·6	3	41,030	6,155	2,175
	Taita Taveta	Yes	62·5	8·7	13·9	1	10,858	1,629	945
	Lamu		47·3	7·3	15·4	3	5,381	807	393
	Mombasa		82·8	13·3	16·1	18	49,198	7,380	6,543
Eastern	Marsabit	Yes	25·8	2·3	8·9	2	8,744	1,312	201
	Kitui	Yes	46·2	5·4	11·7	5	42,087	6,313	2,273
	Isiolo		43·8	6·9	15·8	2	8,168	1,225	564
	Machakos		63·4	11·4	18·0	7	47,372	7,106	5,400
	Makueni		54·6	9·9	18·1	4	34,112	5,117	3,377
	Tharaka-Nithi		76·6	14·5	18·9	4	13,549	2,032	1,965
	Embu		81·5	15·9	19·5	6	19,314	2,897	3,071
	Meru		82·8	18·5	22·3	9	49,678	7,452	9,190
N· Eastern	Mandera	Yes	38·7	1·8	4·7	3	14,894	2,234	268
	Wajir	Yes	21·7	1·3	6·0	4	24,791	3,719	322

For peer review only - http://bmjopen.bmj.com/site/about/guidelines.xhtml

	Garissa	Yes	39·8	5·5	13·8	21	22,773	3,416	1,253
Nyanza	Homa Bay	Yes	60·4	3·1	5·1	5	63,227	9,484	1,960
	Siaya	Yes	70·4	4·3	6·1	4	37,571	5,636	1,616
	Nyamira	Yes	74·1	5·2	7·0	1	18,737	2,811	974
	Kisii	Yes	72·8	5·3	7·3	13	46,315	6,947	2,455
	Kisumu	Yes	69·2	6·8	9·8	14	48,045	7,207	3,267
	Migori	Yes	53·4	6·5	12·2	5	54,290	8,144	3,529
Rift Valley	Turkana	Yes	22·8	0·5	2·2	4	33,343	5,001	167
	Elgeyo Marakwet	Yes	65·0	3·5	5·4	6	16,143	2,421	565
	Nandi	Yes	46·8	2·8	6·0	4	38,628	5,794	1,082
	Uasin Gishu	Yes	59·0	4·8	8·1	10	46,411	6,962	2,228
	West Pokot	Yes	27·0	2·2	8·1	2	29,019	4,353	638
	Bomet	Yes	52·2	4·8	9·2	8	48,237	7,236	2,315
	Baringo	Yes	53·8	5·2	9·7	4	22,869	3,430	1,189
	Nakuru	Yes	69·5	6·9	9·9	11	87,345	13,102	6,027
	Samburu	Yes	29·0	2·9	10·0	3	11,242	1,686	326
	Narok	Yes	40·3	5·1	12·7	5	61,305	9,196	3,127
	Trans-Nzoia	Yes	41·8	5·3	12·7	3	50,735	7,610	2,689
	Laikipia	Yes	49·5	6·5	13·1	3	20,083	3,012	1,305
	Kericho		64·4	10·3	16·0	9	35,841	5,376	3,692
	Kajiado		63·2	11·1	17·6	8	44,297	6,645	4,917
Western	Kakamega	Yes	48·6	3·1	6·4	6	71,779	10,767	2,225
	Busia	Yes	58·5	4·0	6·8	7	39,301	5,895	1,572
	Bungoma	Yes	41·4	5·0	12·1	7	83,598	12,540	4,180
	Vihiga	Yes	50·3	6·6	13·1	4	22,004	3,301	1,452
Nairobi	Nairobi		89·1	20·7	23·2	51	197,079	29,562	40,795
**KENYA**		**57**	**61·8**	**8·7**	**14·0**	**340**	**1,879,796**	**281,969**	**162,828**
Target Counties		*33*	*52·9*	*4·9*	*9·3*	*191*	*1,256,657*	*188,498*	*61,459*

CS = caesarean delivery procedure

For peer review only - http://bmjopen.bmj.com/site/about/guidelines.xhtml

TABLE S2· ESM-KETAMINE PROGRAM SCALE UP TARGETS

Year	1	2	3	4	5
ESMK providers trained initially	72	144	144	144	69
ESMK provider trained to offset turnover	5	15	25	35	40
Total ESMK providers in service	72	216	360	504	**573**
Absolute number of QA/QC staff	1	2	2	3	4
Facilities "on-line"	24	72	120	168	**191**
Coverage	13%	38%	63%	88%	100%

ESMK= Every Second Matters -Ketamine program, QA/QC = Quality Assurance/ Quality Control

TABLE S3· ESM-KETAMINE PROGRAM COST PROJECTION· BASE CASE, UNDISCOUNTED

Year	1	2	3	4	5	Total
Training (initial)	$61,200	$122,400	$122,400	$122,400	$58,650	$487,050
Training (replacement)	$4,250	$12,750	$21,250	$29,750	$34,000	$102,000
QA/QC	$16,400	$32,800	$32,800	$49,200	$65,600	$196,800
Coordination	$48,000	$48,000	$48,000	$48,000	$48,000	$240,000
Overhead	$19,478	$32,393	$33,668	$37,403	$30,938	$153,880
Total	$149,328	$248,343	$258,118	$286,753	$237,188	**$1,179,730**

QA/QC = Quality Assurance/Quality Control

For peer review only - http://bmjopen.bmj.com/site/about/guidelines.xhtml

FIG S1. ANNUAL COST OF SCALING UP ESM-KETAMINE PROGRAM, BY ACTIVITY

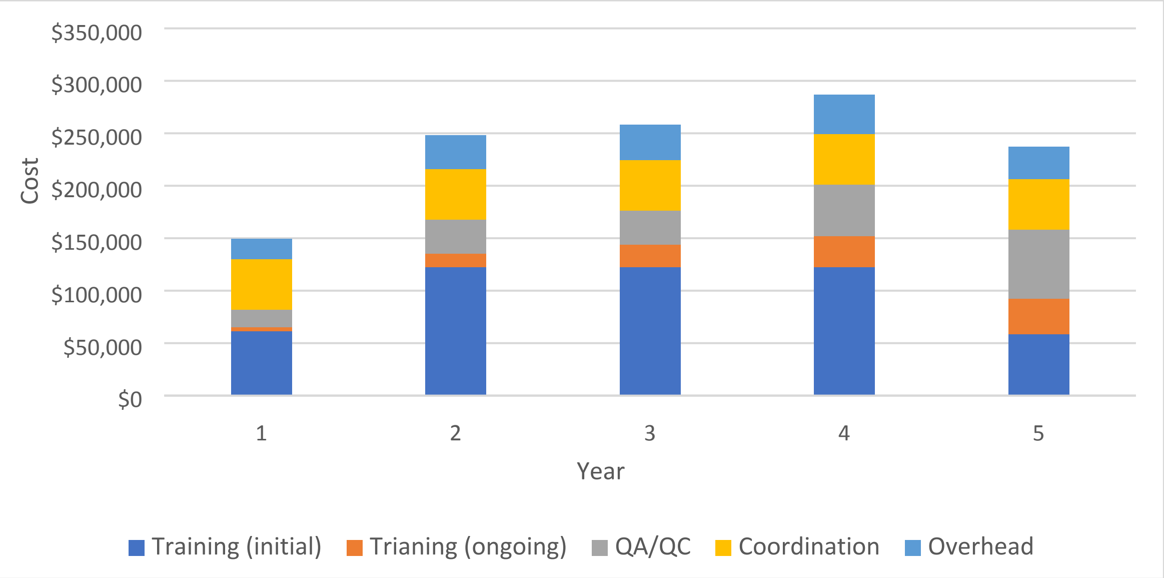

QA/QC = Quality Assurance/Quality Control

TABLE S4. EXPECTED HEALTH IMPACT OF EMERGENCY CAESAREAN DELIVERY, WEIGHTED AVERAGE LIVES SAVED ACROSS ALL INDICATIONS.

	Lives Saved	Lives saved per Caesarean delivery	Number of procedures to save 1 life
Maternal	154	0·011	92
Foetal	2,339	0·165	6·1
Total	2,493	0·178	5·7

For peer review only - http://bmjopen.bmj.com/site/about/guidelines.xhtml

TABLE S5. FOETAL AND MATERNAL DEATHS IN 2017 ATTRIBUTABLE TO COMPLICATIONS THAT INDICATE EMERGENCY CAESAREAN DELIVERY UNDER A BASELINE SCENARIO AND AN "IMPROVED" SCENARIO IN WHICH THERE ARE NO BOTTLENECKS TO EMERGENCY CAESAREAN DELIVERY IN HOSPITAL FACILITIES.

Complication	Foetal Deaths: Baseline	Foetal Deaths: Improved	Foetal Deaths Prevented	Percent of Foetal Deaths Prevented
Foetal Abruption	5,093	4982	111	5%
Foetal Previa	508	494	14	1%
Foetal Ruptured Uterus	1,618	1554	64	3%
Foetal PE/E	6,077	5712	365	16%
SFD MB	488	461	28	1%
SFD IUGR	4,899	4635	264	11%
SFD Breech	498	470	27	1%
SFD Cord	1,996	1889	108	5%
SFD De Novo	5,891	5575	316	14%
Foetal OL	7,195	6665	530	23%
NN Sepsis Term	6,490	6395	95	4%
Term BA	12,318	11900	418	18%
Total Foetal Deaths	53,071	50732	2,339	100%

Complication	Maternal Deaths: Baseline	Maternal Deaths: Improved	Maternal Deaths Prevented	Percent of Maternal Deaths Prevented
Maternal Sepsis	731	717	14	9%
Abruption	348	337	12	8%
Previa	104	100	4	3%
Ruptured Uterus	216	205	11	7%
Atonic Uterus	2,262	2,254	8	5%
PE/E	931	825	106	69%
Total Maternal Deaths	4,592	4,439	154	100%
**Total Deaths**	**57,663**	**55,171**	**2,493**	

For peer review only

For peer review only - http://bmjopen.bmj.com/site/about/guidelines.xhtml

BMJ Open

Non-anaesthetist Administered Ketamine for Emergency Caesarean Section in Kenya: Cost-effectiveness Analysis

Journal:	*BMJ Open*
Manuscript ID	bmjopen-2021-051055.R1
Article Type:	Original research
Date Submitted by the Author:	17-Jun-2022
Complete List of Authors:	Resch, Stephen; Harvard University T H Chan School of Public Health, Center for Health Decision Science<br>Suarez, Sebastian ; Massachusetts General Hospital; Boston University<br>Omotayo, Moshood Olanrewaju ; Massachusetts General Hospital; Harvard Medical School<br>Griffin, Jennifer; RTI International, Center for Global Health<br>Sessler, Daniel; Cleveland Clinic, Department of Outcomes Research<br>Burke, Thomas; Harvard Medical School, Division of Global Health and Human Rights, Department of Emergency Medicine, Massachusetts General Hospital; Harvard University T H Chan School of Public Health
**Primary Subject Heading**:	Anaesthesia
Secondary Subject Heading:	Global health, Health economics
Keywords:	Health economics < HEALTH SERVICES ADMINISTRATION & MANAGEMENT, Anaesthesia in obstetrics < ANAESTHETICS, PUBLIC HEALTH

SCHOLARONE™
Manuscripts

I, the Submitting Author has the right to grant and does grant on behalf of all authors of the Work (as defined in the below author licence), an exclusive licence and/or a non-exclusive licence for contributions from authors who are: i) UK Crown employees; ii) where BMJ has agreed a CC-BY licence shall apply, and/or iii) in accordance with the terms applicable for US Federal Government officers or employees acting as part of their official duties; on a worldwide, perpetual, irrevocable, royalty-free basis to BMJ Publishing Group Ltd ("BMJ") its licensees and where the relevant Journal is co-owned by BMJ to the co-owners of the Journal, to publish the Work in this journal and any other BMJ products and to exploit all rights, as set out in our licence.

The Submitting Author accepts and understands that any supply made under these terms is made by BMJ to the Submitting Author unless you are acting as an employee on behalf of your employer or a postgraduate student of an affiliated institution which is paying any applicable article publishing charge ("APC") for Open Access articles. Where the Submitting Author wishes to make the Work available on an Open Access basis (and intends to pay the relevant APC), the terms of reuse of such Open Access shall be governed by a Creative Commons licence – details of these licences and which Creative Commons licence will apply to this Work are set out in our licence referred to above.

Other than as permitted in any relevant BMJ Author's Self Archiving Policies, I confirm this Work has not been accepted for publication elsewhere, is not being considered for publication elsewhere and does not duplicate material already published. I confirm all authors consent to publication of this Work and authorise the granting of this licence.

For peer review only

Non-anaesthetist administered Ketamine for Emergency Caesarean Section in Kenya: Cost-effectiveness Analysis

Stephen C. Resch, Ph.D., M.P.H. Harvard TH Chan School of Public Health, Boston, MA

Sebastian Suarez, M.D. M.P.H. Massachusetts General Hospital and Boston University, Boston, MA

Moshood Olanrewaju Omotayo, Ph.D. Massachusetts General Hospital and Harvard Medical School, Boston, MA

Jennifer B. Griffin, Ph.D., MPH Center for Global Health, RTI International, Research Triangle Park, NC

Daniel I. Sessler, M.D., Department of Outcomes Research, Cleveland Clinic, Cleveland, OH

Thomas F. Burke, M.D., Department of Emergency Medicine, Massachusetts General Hospital; Harvard Medical School; and Harvard TH Chan School of Public Health, Boston, MA

Corresponding Author: Stephen C. Resch, sresch@hsph.harvard.edu, +1(617) 432-1566

Center for Health Decision Science, Harvard T.H. Chan School of Public Health, 718 Huntington Avenue, Boston MA 02115

Article Type: Original Research

Word Count: Abstract – 250, Main Text -- 3309

Synopsis:

Scaling up a program to train clinicians to administer ketamine as anaesthesia is a cost-effective approach to enabling life-saving emergency caesarean deliveries in Kenya.

For peer review only - http://bmjopen.bmj.com/site/about/guidelines.xhtml

Abstract

Objectives: Lack of anaesthesia services is a frequent barrier to emergency surgeries such as caesarean delivery in Kenya. This study aimed to estimate the survival gains and cost-effectiveness of scaling up the Every Second Matters (ESM) – Ketamine program that trains non-anaesthetist providers to administer and monitor ketamine during emergency caesarean deliveries.

Setting: Hospitals in Kenyan counties with low rates of caesarean delivery.

Participants: Patients needing emergency caesarean delivery in settings without availability of standard anaesthesia service.

Interventions: Simulated scales up of the ESM-Ketamine program over five years (2020-24) was compared to status quo.

Outcome measures: Cost of implementing the program and corresponding additional emergency caesarean deliveries. Maternal and foetal/neonatal deaths prevented, and corresponding life-years gained due to increased provision of emergency caesarean procedures. Cost-effectiveness was assessed by comparing the cost per life-year gained of the ESM-Ketamine program compared to status quo.

Results: Over five years, the expected gap in emergency caesarean deliveries was 157,000. A $1.2 million ESM-Ketamine program reduced this gap by 28,700, averting by 316 maternal and 4736 foetal deaths and generating 331,000 total life-years gained. Cost-effectiveness of scaling up the ESM-Ketamine program was $44 per life year gained in the base case and $251 in the most pessimistic scenario—a very good value for Kenya at less than 20% of per capita GDP per life-year gained.

Conclusion: In areas of Kenya with significant under-provision of emergency caesarean delivery due to a lack of availability of traditional anaesthesia, an ESM-Ketamine program is likely to enable a substantial number of life-saving surgeries at reasonable cost.

For peer review only - http://bmjopen.bmj.com/site/about/guidelines.xhtml

Strengths and limitations of this study

- This study uses a decision analytic approach that allows synthesis of data on costs and outcomes from a long-running ESM-Ketamine program implemented in one county in Kenya and use a simulation model to extrapolate the impact and cost-effectiveness of expanding the program throughout the country.
- Another strength of this method is that we are able to translate the observed emergency caesarean deliveries enabled by the ESM-Ketamine program into maternal and foetal deaths averted and life-year gains using the MANDATE model to simulate outcomes of deliveries with complications when surgery is available and for a counterfactual scenario where surgery is not available.
- A limitation of this study is the uncertainty about the extent to which lack of available anaesthesia is the key barrier to timely provision of emergency surgeries in Kenyan health facilities.
- The study is also limited by uncertainty regarding the pace at which the supply of conventional anaesthesiology services can be expanded, such that the ESM-Ketamine program would no longer be needed.
- The study was limited to considering impact on childbirth-related mortality and did not capture potential additional benefits related to reductions in morbidity or mortality from other causes requiring emergency surgery.

For peer review only - http://bmjopen.bmj.com/site/about/guidelines.xhtml

Introduction

The World Health Organization (WHO) estimates that at least 10-15% and possibly as many as 19%[1,2] of all childbirths require caesarean deliveries, most on an emergency basis. Few developing countries provide this level of caesarean deliveries, with considerable consequent morbidity and mortality. Kenya provides more caesarean deliveries than most sub-Saharan African countries, with a population rate of 8.7% [3,4]. However, this average rate masks substantial heterogeneity. In 16 of 47 counties the population caesarean delivery rate is below 5%. In 26 counties the rate is between 5% and 15%. In just five counties, mostly those with large urban areas such as Nairobi, the population caesarean delivery rate is over 15%. While rigorous data is lacking, a substantial portion of caesarean procedures contributing to the rate observed in urban areas may be elective [5].

Undersupply of emergency caesarean deliveries in many parts of Kenya likely contributes to the country's high maternal and perinatal mortality. When last measured in 2014, the maternal mortality ratio (MMR) in Kenya was 362 (95% CI: 254, 471) per 100,000 births [4]. Perinatal mortality, defined as pregnancy losses occurring after seven completed months of gestation (stillbirths) plus deaths to live births within the first seven days of life (early neonatal deaths), was 29 per 1000 births in 2014 [4,6].

One of the major limitations to provision of caesarean deliveries is lack of anaesthesia. The Lancet Commission on Global Surgery indicated substantially improved health outcomes associated with increases in specialist surgical workforce (surgeons, anaesthetists, and obstetricians) up to at least 20 per 100,000 population, and the

For peer review only - http://bmjopen.bmj.com/site/about/guidelines.xhtml

World Federation of Societies of Anaesthesiologists has indicated about half of these providers – i.e. 10 per 100,000 population—should be anaesthesia providers [7,8]. In Kenya, there are less than 2 anaesthesia providers per 100,000 population, of which about 80% are non-physician anaesthetists including clinical officers and nurses [7]. The lack of anaesthesia services for emergency surgery is especially grave when considering that the workforce is maldistributed within the country in proportion to the population [9].

We have previously described the Every Second Matters for Emergency and Essential Surgery – Ketamine (ESM-Ketamine) program which has been operating in rural Kenya since 2013 [10,11]. While not envisioned as a long-term solution to the shortage of anaesthesia services in Kenya, or a substitute for other health system strengthening efforts aimed at increasing the supply and improving the distribution of anaesthetists and anaesthesiologists, the ESM-Ketamine program was designed as a rapid, low-cost approach to ensuring anaesthesia is available for surgery in emergency situations. Through this program, non-anaesthetist providers are trained to administer and monitor ketamine during emergency surgeries such as caesarean delivery when no anaesthetist is available, enabling procedures that would have not been possible, would have been significantly delayed, or would have been performed without any anaesthesia. The program includes an intensive hands-on five-day training, ESM-Ketamine kits, wallcharts, checklists, and regular supervisory visits for quality assurance. The ESM-Ketamine pilot program in Kenya has proven remarkably safe, with positive patient experiences, no deaths or major adverse events attributed to ketamine in more than 2,000 emergency and essential surgeries including about 450 caesarean deliveries

For peer review only

For peer review only - http://bmjopen.bmj.com/site/about/guidelines.xhtml

across 17 hospitals [12,13]. However, there are distinct training, implementation, and running costs associated with the ESM-Ketamine program. In this analysis, we model the potential health impact and cost-effectiveness of scaling up the ESM-Ketamine program for emergency caesarean sections throughout Kenya as compared to the status quo.

Methods

Our decision analytic approach, shown in Figure 1, synthesized data from numerous sources to simulate the health impact and cost of scaling up the ESM-Ketamine program nationally in Kenya. Using data from the 2014 Kenya Demographic and Health Survey [4], we estimated the facility caesarean delivery rate (caesarean delivery performed divided by deliveries with a skilled provider) for all counties in Kenya and identified those where the facility caesarean delivery rate was less than 15%. For these counties, we estimated the number of facility-based deliveries over a five-year period (2020-2024) using UN projections of annual births and calculated the county-specific gap in emergency caesarean delivery using an expected unconstrained emergency caesarean delivery rate of 15% as the reference level.

Ketamine-based anaesthesia via providers trained in the ESM-Ketamine program addresses only one of several possible bottlenecks to emergency caesarean delivery. Thus, the impact of the program will depend on the frequency with which the availability of traditional anaesthesia is the sole bottleneck. While no studies have measured this precisely at a patient case level, there is evidence that in some settings in Kenya, anaesthesia service is in shorter supply than other necessary service components for

For peer review only - http://bmjopen.bmj.com/site/about/guidelines.xhtml

emergency caesarean. A 2011 study in Nyanza region found that for 34 operating theatres with associated doctors able to perform caesarean delivery, there were only 18 anaesthetists and 44% of theatres reported severely limited access to anaesthetist services[14]. More recently, in 2014, we surveyed all 30 operating theatres in 2 counties of Western Kenya and found 57% had no access to anaesthesia services [15]. Based on this evidence, we assumed that anaesthesia availability was the sole bottleneck in 30% of the cases in which an emergency caesarean delivery could not be provided.

We modelled a gradual five-year scale up of the ESM-Ketamine program that reaches full coverage of all facilities with operating rooms in the target counties (Table 1). Based on the level of scale to be achieved each year, the number of additional emergency caesarean deliveries was calculated for each of the five years as compared to a status quo with no change in caesarean availability.

We used the previously developed, publicly-available MANDATE model (www.mandate4mnh.org) to estimate the expected number of lives saved per additional emergency caesarean delivery in Kenya. This model synthesizes evidence on the incidence, case fatality rate, and efficacy of caesarean delivery for all major delivery complications for which emergency caesarean delivery is the recommended intervention[16,17]. We simulated two scenarios with this model: a base case approximating status quo for Kenya in 2017 and an alternative case where caesarean delivery is available (and used when indicated) in 99% of emergency cases in hospital settings. See Table S1 for a detailed list of the baseline penetration and utilization rates of Caesarean section for each condition, as well as the corresponding efficacy parameters. We calculated the difference in caesarean delivery procedures performed

For peer review only - http://bmjopen.bmj.com/site/about/guidelines.xhtml

and the difference in foetal and maternal deaths, and then calculated a ratio of deaths prevented per additional caesarean delivery procedure. We applied these ratios to the number of additional caesarean deliveries we estimated would occur in Kenya each year because of the scale-up of the ESM-Ketamine program.

We converted deaths averted to years of life gained using the most recently available 2016 Kenya life tables. For foetal deaths prevented, we used the average of male and female life expectancy at less than one year of age. For maternal deaths prevented, we used the average of remaining life expectancy for three female age groups (20-24, 25-29, 30-34 years).

Evaluation of the ESM-Ketamine program in 2059 Kenyan patients identified only occasional mild adverse events associated with Ketamine such as hallucinations and salivation, and no major adverse events [12]. We therefore assumed that ESM-Ketamine does not cause major morbidities and did not include the minor consequences of the occasional mild and time-limited adverse events since they pale in comparison to the consequences of not providing emergency caesarean delivery.

Costs

Based on the experience of the current ESM-Ketamine program in Kenya, we modelled the health-sector cost of scaling up access. We assumed three ESM-Ketamine trained providers would be required in each facility with surgical capacity to ensure that availability of anaesthesia services would not be a bottleneck to emergency caesarean delivery. In the first year, 72 providers are expected to graduate from the training program. In subsequent years, a second training centre is added, and the number of

For peer review only

For peer review only - http://bmjopen.bmj.com/site/about/guidelines.xhtml

providers trained per year increases to 144. In the fifth year, the number of newly trained reduces as the necessary supply of ESM-Ketamine providers is reached. We assumed a 7% annual staff turnover rate and included additional training to maintain target supply of active ESM-Ketamine providers. The unit cost of training one provider was $650 plus $150 for travel and lodging. In addition, each facility requires a $150 ESM-Ketamine kit (Table 1 and S2). We included cost for supervision and coordination, quality assurance (QA) activities, and overhead. These costs included a full-time program director, one quality assurance (QA) officer per 60 facilities, and 15% overhead.

The cost of additional caesarean delivery procedures ($367 per delivery) was based on a recent rigorous cost analysis conducted in Rwanda adjusted for Kenya based on differences in purchasing power parity [18]. Because some of cost would be incurred even if no caesarean procedure was performed, we subtracted the cost of a vaginal delivery – which we assumed was half the cost of a caesarean delivery [19].

Cost-effectiveness Analysis

To calculate the incremental cost per life year gained, we divided the five-year program cost (in 2017 US dollars) by the number of life years gained due to lives saved in the five-year period, discounting both costs and health outcomes by 3% per year. We also reported undiscounted results. Kenya's GDP per capita in 2018 was US$1620 [20]. Considerable debate persists about value thresholds for health interventions in settings like Kenya. The WHO has historically argued that interventions with an ICER less than per-capita GDP per DALY averted are cost-effective. More recently, researchers have

For peer review only - http://bmjopen.bmj.com/site/about/guidelines.xhtml

advocated that much more stringent health-opportunity-loss based thresholds, are more appropriate when deciding to invest in new health technologies where health budgets are fixed [21]. Under this approach, interventions costing less than 25% of GDP per capita per disability-adjusted life-year (DALY) averted are generally considered highly cost-effective by most standards [22]. Our model estimated life-years gained but could not account for disability in those years. Therefore, we used 20% GDP per capita per life year gained ($325 in Kenya) as a benchmark for good value, which is approximately equivalent to assuming an average lifetime health-related quality of life of 0.85 (where one represents perfect health and zero represents death) [23].

Patient and Public Involvement

Our study uses aggregated secondary data from the ESM-Ketamine program in Kenya regarding outcomes for patients undergoing surgery with Ketamine anaesthesia. But there is no patient involvement in this model-based cost-effectiveness study.

Ethical Clearance

This model-based study did not collect any new information from human subjects or use any patient-level data, so we did not obtain approval of an Institutional Review Board. Neither patients nor the public were not involved in the design, conduct, reporting, or dissemination plans of our research.

Data Availability

No additional data available.

For peer review only - http://bmjopen.bmj.com/site/about/guidelines.xhtml

Sensitivity Analysis

The impact and value of the ESM-Ketamine program will depend on the actual number of emergency caesarean deliveries that are enabled by the program; which in turn depends both on the number of emergency cases requiring caesarean delivery that are currently not receiving caesarean delivery as well as the portion of these cases in which lack of anaesthesia is the sole bottleneck preventing the caesarean delivery procedure from being performed. In sensitivity analysis, we considered scenarios in which only 10% of all hospital deliveries require emergency caesarean delivery (vs. 15% rate assumed in base case), and scenarios in which lack of anaesthesia was the sole bottleneck for only 10% of cases in which emergency caesarean delivery was indicated but not provided (vs. 30% in the base case). We also considered the case in which the ESM-Ketamine program costs and the additional delivery costs associated with caesarean section were twice as high as in the base case.

For peer review only

Results

The results are reported according to the specifications of the Consolidated Health Economic Evaluation Reporting Standards statement as documented in the checklist (Table S2).

Program scope and cost

In Kenya, 33 counties met the criteria of having a facility caesarean delivery rate less than 15% (Table S3). The emergency caesarean delivery gap across these counties was 157,000 procedures over five years, of which 47,000 are attributable to lack of anaesthesia. The target counties contain 191 hospitals with operating rooms. The modelled ESM-Ketamine program would train 693 providers, enough to maintain 3 providers per facility, while accounting for staff turnover (Table S4). The scale up of the ESM-Ketamine program from 13% coverage in Year 1 to 100% coverage in Year 5 would enable 28,700 emergency caesarean deliveries, reducing the overall gap in emergency caesarean delivery by 18% and the anaesthesia-attributable gap by 61%. The undiscounted cost of the five-year scale up of the ESM-Ketamine program is estimated to be about $1.2 million (Table S5), or $1700 per ESM-Ketamine provider trained. About half of this cost is the direct cost of training providers (Figure S1). The cost of the ESM-Ketamine program would be and $41 per emergency caesarean delivery enabled. The total incremental cost, when including the additional cost of the caesarean procedure itself, is about $224 per delivery.

For peer review only - http://bmjopen.bmj.com/site/about/guidelines.xhtml

Health Outcomes

190 Results from the MANDATE model indicate that the most common indications for
191 emergency caesarean delivery are obstructed labour, pre-eclampsia or eclampsia, and
192 foetal distress (Table 2). In the baseline scenario, the hospital caesarean delivery rate
193 was 7.1%. By maximizing the availability and use of caesarean delivery for emergency
194 indications in hospital settings (without changing care-seeking patterns or the
195 effectiveness of the emergency medical transport system) the number of caesarean
196 deliveries almost doubles—to a rate of 12.3% – which corresponds well to the level of
197 emergency caesarean delivery expected based on the incidence of life-saving
198 indications found in an observational study [24].

199 Analysing the corresponding reduction in maternal and foetal death for improved access
200 to caesarean delivery compared to the status quo, we found that 0.178 deaths would be
201 averted per additional emergency caesarean procedure provided (Table 2 and S6).
202 Over 90% of these deaths were foetal. One foetal death was prevented by every 6.1
203 emergency caesarean delivery procedures. One maternal death was averted by every
204 92 emergency caesarean delivery procedures. These estimates represent a weighted
205 average across the emergency indications and accounts for condition-specific mortality
206 risk reduction conferred by caesarean delivery (Table S7).

207 Applying these rates of life-saving to the additional 28,700 caesarean deliveries
208 provided over five years as a result of the scale-up of the ESM-Ketamine program, we
209 estimated that maternal and foetal deaths would be reduced by 316 and 4736

For peer review only - http://bmjopen.bmj.com/site/about/guidelines.xhtml

respectively (Table 3). Considering remaining life expectancies at the moment these deaths are averted, the mortality reductions would translate into 331,000 total life-years gained.

Cost-effectiveness

Cost-effectiveness results are shown in Table 4. Without discounting, the average cost per death prevented by ketamine-enabled caesarean delivery performed during the five-year period in the base case was about $1,270. Considering the expected years of life gained by preventing deaths, and discounting both costs and future life-years, the cost-effectiveness of scaling up the ESM-Ketamine program was $44 per life year gained.

In sensitivity analysis, when the caesarean delivery gap among hospital deliveries is based on a bottleneck-free caesarean delivery rate of 10%, and the portion of the gap attributable to lack of anaesthesia is only 10%, an ESM-Ketamine program would prevent 28 maternal and 426 foetal deaths, translating to 29,750 life years gained, and a cost-effectiveness of $125 per life-year gained. In the most pessimistic scenario considered, combining lower impact on caesarean section uptake, higher caesarean procedure cost, and a doubling of the cost of the ESM-Ketamine program itself, the cost-effectiveness ratio would increase to $251 per life year gained.

Discussion

Our analysis shows an ESM-Ketamine program could enable an additional 28,700 emergency caesarean deliveries over five years, reducing the projected gap in emergency caesarean deliveries in 33 Kenyan counties by about 18%. The cost of the

For peer review only - http://bmjopen.bmj.com/site/about/guidelines.xhtml

ESM-Ketamine program would be about $1700 per ESM-Ketamine provider trained and $41 per emergency caesarean delivery enabled. The total incremental cost, when including the additional cost of the caesarean procedure itself, is about $224 per delivery.

Given that we found one death is expected to be prevented by every six emergency caesarean delivery procedures, the program is likely to be highly cost-effective. In the base case, the scale up of ESM-Ketamine program over five years had a net cost of $5.9 million and resulted in 5052 deaths averted, translating to an incremental cost-effectiveness ratio of $44 per life year gained.

There is substantial uncertainty in the data that underlies our analysis, which could limit confidence in the results. The impact of the ESM-Ketamine program on clinical outcomes, compared to a status quo counterfactual, has not been measured in a randomized controlled trial. Therefore, we modelled the number of emergency caesarean procedures enabled by ESM-Ketamine, as well as the clinical outcomes for deliveries requiring caesarean when ketamine is available and when it is not. To address the uncertainty in our model, we tested the sensitivity of our conclusions about the value of the ESM-Ketamine program over a wide range of less favourable assumptions. In the most pessimistic scenario we considered – in which the number of caesarean deliveries enabled was reduced to 2580 (9% of the base case amount) and the cost of both the ESM-program itself and the cost of caesarean deliveries was doubled, the cost per life year gained only increased to $251. In all scenarios, the cost-effectiveness ratios were far less than 20% of GDP per capita ($325) benchmark we used as a threshold value and compare favourably to many public health interventions

For peer review only - http://bmjopen.bmj.com/site/about/guidelines.xhtml

254 being implemented in Kenya. Indeed, using $325 per life year as a threshold for good
255 value, the ESM-Ketamine program would only have to enable 1720 emergency
256 caesarean deliveries and avert about 300 deaths in five years to be considered cost-
257 effective — which is only about 6% of the impact we estimate.

258 Our analysis assumed that there would be no change in the portion of deliveries in
259 health facilities, no change in the availability of timely emergency transport to hospitals
260 with capacity for caesarean delivery, and no reduction in the caesarean delivery gap
261 due to increasing availability of traditional anaesthesia services. If more deliveries were
262 to occur in health facilities over time, then there would be more opportunities for the
263 ESM-Ketamine program to enable life-saving emergency caesarean delivery. If the
264 expansion of traditional anaesthesia service reduces the gap in caesarean delivery, the
265 ESM-Ketamine program would be less cost-effective and, ideally, unnecessary.
266 However, the payback period for an ESM-Ketamine program is very short. It is highly
267 unlikely that the investment in establishing a program and training ESM-Ketamine
268 providers would be rendered moot by a sudden expansion of traditional anaesthesia
269 services. For example, in the base case, each ESM-Ketamine trained provider would
270 enable about 41 emergency caesarean deliveries on average over the five-year time
271 horizon, resulting in about 7.3 lives saved per ESM-Ketamine provider trained. Yet, to
272 meet standard benchmarks for cost-effectiveness, each ESM-Ketamine provider would
273 only need to enable about three emergency caesarean deliveries — a number that
274 might reasonably be expected within a few months of completing training.

275 The expected cost of the ESM-Ketamine program is small relative to the cost of
276 caesarean delivery procedures enabled by the availability of ketamine anaesthesia.

For peer review only - http://bmjopen.bmj.com/site/about/guidelines.xhtml

Therefore, the value of the ESM-Ketamine program is driven in large part by the value of emergency caesarean delivery itself. The overall cost-effectiveness of ESM-Ketamine was much more sensitive to the additional cost of caesarean delivery compared to vaginal delivery than the cost of the ESM-Ketamine program itself.

Another limitation in our analysis is the scope of benefits considered. Although caesarean delivery is also likely to prevent morbidity in both mothers and new-borns (e.g., long-term cognitive problems associated with non-fatal birth asphyxia), we did not include these health benefits. We also did not consider the value of the ESM-Ketamine program related to surgical procedures other than emergency caesarean deliveries. In the ESM-Ketamine program in Kenya, emergency caesarean deliveries account for about 20% of the total procedures performed with many of the remaining procedures being for acute abdomens, open fractures, and similarly serious conditions. For these reasons, it is likely that the total value of the ESM-Ketamine program is larger than we estimated.

Our analysis supports the relatively modest investment needed for scaling up the ESM-Ketamine program over the next five years. Cost per life year gained ranged from $44 (2.8% of GDP per capita) in the base case to $251 (16% of GDP per capita) in our most pessimistic scenario. There remains significant uncertainty about the overall scale of the impact of an ESM-Ketamine program due to a lack of data regarding the extent to which anaesthesia is a key bottleneck preventing access to emergency caesarean delivery. Nevertheless, we found that the ESM-Ketamine program would be worthwhile even if the number of emergency caesarean procedures it enabled were an order-of-magnitude smaller than expected in our base case. In areas of Kenya with significant under-

For peer review only - http://bmjopen.bmj.com/site/about/guidelines.xhtml

provision of emergency caesarean delivery due to a lack of availability of traditional anaesthesia, an ESM-Ketamine program is likely to enable a substantial number of life-saving surgeries at modest cost.

For peer review only

For peer review only - http://bmjopen.bmj.com/site/about/guidelines.xhtml

Author contributions

The study was conceptualized and designed by SR, TB, and SS. SS and MO assisted in gathering information from the ESM-Ketamine pilot project to inform parameters of the cost-effectiveness model. JG conducted analysis with the Mandate model to inform parameters regarding the impact of emergency caesarean section on neonatal and maternal deaths. SR developed the cost-effectiveness model and conducted the analysis. SR, with DS and TB, wrote the manuscript. All authors reviewed and edited manuscript drafts.

Funding information

The work presented in this manuscript was funded by USAID Award No. 7200AA18CA00002 (project title: “Scaling Up Every Second Matters for Mothers and Babies – Ketamine (ESM-Ketamine) in Kenya”) and supported by the Ujenzi Charitable Trust (Grant number: Not Applicable) , Elrha’s Research for Health in Humanitarian Crises (R2HC) Program (Grant number: Not Applicable) and the Saving Lives at Birth partners (Grant number: Not Applicable). The Saving Lives at Birth partners are: United States Agency for International Development (USAID), the Government of Norway, the Bill & Melinda Gates Foundation, Grand Challenges Canada, the UK Government, and the Korea International Cooperation Agency (KOICA). The R2HC program is funded by the UK Foreign, Commonwealth and Development Office (FCDO), Wellcome, and the UK National Institute for Health Research (NIHR). This manuscript was prepared by the listed authors and does not necessarily reflect the views of the funding partners. The

For peer review only - http://bmjopen.bmj.com/site/about/guidelines.xhtml

funding sources had no role in the collection, analysis, or interpretation of the data nor in manuscript preparation.

Competing interests

The authors have no conflicts of interest.

For peer review only

References

1. Molina G, Weiser TG, Lipsitz SR, Esquivel MM, Uribe-Leitz T, Azad T, et al. Relationship between cesarean delivery rate and maternal and neonatal mortality. JAMA. 2015;314: 2263–2270. doi:10.1001/jama.2015.15553

2. Betran AP, Torloni MR, Zhang JJ, Gülmezoglu AM, WHO Working Group on Caesarean Section. WHO statement on caesarean section rates. BJOG. 2016;123: 667–670. doi:10.1111/1471-0528.13526

3. Harrison MS, Goldenberg RL. Cesarean section in sub-Saharan Africa. Matern Health Neonatol Perinatol. 2016;2: 6. doi:10.1186/s40748-016-0033-x

4. Kenya National Bureau of Statistics, Ministry of Health/Kenya, National AIDS Control Council/Kenya, Kenya Medical Research Institute, National Council for Population and Development/Kenya, ICF International. Kenya Demographic and Health Survey 2014. Rockville, MD, USA: Kenya National Bureau of Statistics, Ministry of Health/Kenya, National AIDS Control Council/Kenya, Kenya Medical Research Institute, National Council for Population and Development/Kenya, and ICF International; 2015.

5. van der Spek L, Sanglier S, Mabeya HM, van den Akker T, Mertens PLJM, Houweling TAJ. Socioeconomic differences in caesarean section - are they explained by medical need? An analysis of patient record data of a large Kenyan hospital. Int J Equity Health. 2020;19: 117. doi:10.1186/s12939-020-01215-2

For peer review only

For peer review only - http://bmjopen.bmj.com/site/about/guidelines.xhtml

6. Kunkel M, Marete I, Cheng ER, Bucher S, Liechty E, Esamai F, et al. Place of delivery and perinatal mortality in Kenya. Semin Perinatol. 2019;43: 252–259. doi:10.1053/j.semperi.2019.03.014

7. Kempthorne P, Morriss WW, Mellin-Olsen J, Gore-Booth J. The WFSA global anesthesia workforce survey. Anesth Analg. 2017;125: 981–990. doi:10.1213/ANE.0000000000002258

8. Meara JG, Leather AJM, Hagander L, Alkire BC, Alonso N, Ameh EA, et al. Global Surgery 2030: evidence and solutions for achieving health, welfare, and economic development. Lancet. 2015;386: 569–624. doi:10.1016/S0140-6736(15)60160-X

9. Epiu I, Tindimwebwa JVB, Mijumbi C, Chokwe TM, Lugazia E, Ndarugirire F, et al. Challenges of Anesthesia in Low- and Middle-Income Countries: A Cross-Sectional Survey of Access to Safe Obstetric Anesthesia in East Africa. Anesth Analg. 2017;124: 290–299. doi:10.1213/ANE.0000000000001690

10. Burke TF, Suarez S, Sessler DI, Senay A, Yusufali T, Masaki C, et al. Safety and Feasibility of a Ketamine Package to Support Emergency and Essential Surgery in Kenya when No Anesthetist is Available: An Analysis of 1216 Consecutive Operative Procedures. World J Surg. 2017;41: 2990–2997. doi:10.1007/s00268-017-4312-0

11. Burke T, Manglani Y, Altawil Z, Dickson A, Clark R, Okelo S, et al. A Safe-Anesthesia Innovation for Emergency and Life-Improving Surgeries When no Anesthetist is Available: A Descriptive Review of 193 Consecutive Surgeries. World J Surg. 2015;39: 2147–2152. doi:10.1007/s00268-015-3118-1

For peer review only

For peer review only - http://bmjopen.bmj.com/site/about/guidelines.xhtml

12. Burke TF, Nelson BD, Kandler T, Altawil Z, Rogo K, Imbamba J, et al. Evaluation of a ketamine-based anesthesia package for use in emergency cesarean delivery or emergency laparotomy when no anesthetist is available. Int J Gynaecol Obstet. 2016;135: 295–298. doi:10.1016/j.ijgo.2016.06.024

13. Villegas S, Suarez S, Owuor J, Wuyke GM, Nelson BD, Imbamba J, et al. Intraoperative awareness and experience with a ketamine-based anaesthesia package to support emergency and essential surgery when no anaesthetist is available. Afr J Emerg Med. 2019;9: S56–S60. doi:10.1016/j.afjem.2018.07.003

14. Ministry of Public Health and Sanitation [Kenya]. Provincial MMC Task force report, Nyanza Province. Ministry of Public Health and Sanitation [Kenya]; 2011 Apr.

15. Burke TF, Hines R, Ahn R, Walters M, Young D, Anderson RE, et al. Emergency and urgent care capacity in a resource-limited setting: an assessment of health facilities in western Kenya. BMJ Open. 2014;4: e006132. doi:10.1136/bmjopen-2014-006132

16. Jones-Hepler B, Moran K, Griffin J, McClure EM, Rouse D, Barbosa C, et al. Maternal and neonatal directed assessment of technologies (MANDATE): methods and assumptions for a predictive model for maternal, fetal, and neonatal mortality interventions. Glob Health Sci Pract. 2017;5: 571–580. doi:10.9745/GHSP-D-16-00174

17. Harrison MS, Griffin JB, McClure EM, Jones B, Moran K, Goldenberg RL. Maternal Mortality from Obstructed Labor: A MANDATE Analysis of the Ability of

For peer review only

Technology to Save Lives in Sub-Saharan Africa. Am J Perinatol. 2016;33: 873–881. doi:10.1055/s-0036-1571322

18. Odhiambo J, Ruhumuriza J, Nkurunziza T, Riviello R, Shrime M, Lin Y, et al. Health Facility Cost of Cesarean Delivery at a Rural District Hospital in Rwanda Using Time-Driven Activity-Based Costing. Matern Child Health J. 2019;23: 613–622. doi:10.1007/s10995-018-2674-z

19. Kabale N. It now costs Sh200,000 to deliver in private hospital. In: It now costs Sh200,000 to deliver in private hospital [Internet]. 4 Jul 2018 [cited 13 Oct 2019]. Available: https://www.businessdailyafrica.com/news/It-now-costs-Sh200-000-to-deliver-in-private-hospital/539546-4645660-wq4fjez/index.html

20. World Bank. World Development Indicators Databank. In: https://data.worldbank.org/indicator/SP.DYN.LE00.IN [Internet]. 2018 [cited 4 Dec 2018]. Available: https://data.worldbank.org/indicator/SP.DYN.LE00.IN

21. Sculpher M, Claxton K, Pearson SD. Developing a value framework: the need to reflect the opportunity costs of funding decisions. Value Health. 2017;20: 234–239. doi:10.1016/j.jval.2016.11.021

22. Woods B, Revill P, Sculpher M, Claxton K. Country-Level Cost-Effectiveness Thresholds: Initial Estimates and the Need for Further Research. Value Health. 2016;19: 929–935. doi:10.1016/j.jval.2016.02.017

For peer review only

For peer review only - http://bmjopen.bmj.com/site/about/guidelines.xhtml

23. Fryback DG, Dasbach EJ, Klein R, Klein BE, Dorn N, Peterson K, et al. The Beaver Dam Health Outcomes Study: initial catalog of health-state quality factors. Med Decis Making. 1993;13: 89–102. doi:10.1177/0272989X9301300202

24. Belizán JM, Minckas N, McClure EM, Saleem S, Moore JL, Goudar SS, et al. An approach to identify a minimum and rational proportion of caesarean sections in resource-poor settings: a global network study. Lancet Glob Health. 2018;6: e894–e901. doi:10.1016/S2214-109X(18)30241-9

For peer review only

TABLE 1. KEY MODEL INPUTS

Parameter	Base case Value	Alternative Values	Source Notes
Demographics			
Estimated deliveries (2020)	1.503 million		UN Population Division (derived from crude birth rate and total population estimate)
Change in total deliveries	0.01		Imputed from UN Population Prospects Births (Medium variant)
Target emergency caesarean delivery rate	15%	10%	
Share of emergency caesarean delivery gap attributable only to lack of anaesthesia services	30%	10%	14, 15
Training Program Implementation Assumptions			
Training centres	1 in first year, 2 thereafter		
Providers trained per session	6		
Trainings per year per training centre	12		
ESMK provider turnover rate	0.07		
ESMK providers per facility	3		
ESM-Ketamine Program Unit Costs			
Training			
Training costs	$650		TTS grant (Jan 2017)
Travel and lodging	$150		MGH / USAID
Kit (1 per facility)	$150		TTS grant
Program Coordination			
Program director	$48,000		TTS grant
QA/QC			

For peer review only - http://bmjopen.bmj.com/site/about/guidelines.xhtml

	Facilities per staff person	60		MGH / USAID
	QA/QC staff salary	$16,400		TTS grant
Overhead		15%		TTS grant
Life expectancy from age x		Male	Female	WHO Life Tables Kenya 2016
	<1 year	64.4	68.9	
	20-24 years		53.3	
	25-29 years		48.8	
	30-34 years		44.4	
Life expectancy per averted death		Undiscounted	Discounted	
	Mothers (average of 3 age groups)	48.8	26.2	
	Newborns (average of Male and Female <1 year)	66.7	29.5	
Discount Rate, annual		3%		
ESMK = Every Second Matters – Ketamine program; QA/QC = Quality assurance/Quality control; TTS =				

For peer review only

For peer review only - http://bmjopen.bmj.com/site/about/guidelines.xhtml

TABLE 2. NUMBER OF EMERGENCY CAESAREAN DELIVERY PROCEDURES BY INDICATION UNDER A BASELINE SCENARIO AND AN "IMPROVED" SCENARIO IN WHICH THERE ARE NO BOTTLENECKS TO EMERGENCY CAESAREAN DELIVERY IN HOSPITAL FACILITIES*

Indication	Baseline	Improved	Difference	Percent of Total Difference
Obstructed Labor	13,214	18,330	5116	36%
Pre-eclampsia / Eclampsia	2241	5469	3228	23%
AIPH - Abruption	495	1280	785	6%
AIPH - Placenta Previa	166	422	256	2%
AIPH - Ruptured Uterus	231	432	201	1%
SFD - MB	73	242	169	1%
SFD - IUGR	698	2327	1629	11%
SFD - Breech	73	241	168	1%
SFD - Cord	284	949	665	5%
SFD - Other	837	2789	1952	14%
Total Procedures	18,312	32,481	14,169	100%
**Deaths Averted**				
Maternal			154	6.2%
Foetal			2339	93.8%
Total			2493	
**Deaths Averted per Emergency Caesarean**				
Maternal			0.011	
Foetal			0.165	
Total			0.178	

*The total number of hospital deliveries was 258,630. In the "baseline" scenario, the emergency caesarean delivery rate is about 7.1% for hospital deliveries, and in the "improved scenario" --representing 99% coverage of emergency caesarean delivery in hospital setting-- the emergency caesarean delivery rate increases to 12.3% of hospital deliveries. In both scenarios, the analysis assumed no non-emergency caesarean deliveries occur for reasons such as maternal request, revenue maximization, or scheduling convenience.

AIPH = Ante/intrapartum Haemorrhage; SFD = Significant foetal distress; MB = multiple births; IUGR = intrauterine growth rate

For peer review only - http://bmjopen.bmj.com/site/about/guidelines.xhtml

TABLE 3. PROGRAMMATIC AND HEALTH OUTCOMES BY YEAR. BASE CASE, UNDISCOUNTED

Year	2020	2021	2022	2023	2024	TOTAL
CS gap	9248	9341	9434	9528	9624	47175
Coverage scale-up	13%	38%	63%	88%	100%	61%
Additional CS provided	1202	3549	5943	8385	9624	28,704
Lives Saved						
Maternal	13	39	65	92	106	316
Foetal	198	586	981	1384	1588	4736
Total	212	625	1046	1476	1694	5052
Life Years Gained						
Maternal	646	1907	3193	4504	5170	15419
New-born	13,222	39,034	65,362	92,212	105,834	315,664
Total	13,867	40,941	68,554	96,716	111,004	331,083
Economic outcomes						
ESMK program	$149,328	$248,343	$258,118	$286,753	$237,188	$1,179,730
Additional CS	$219,791	$648,892	$1,086,553	$1,532,902	$1,759,353	$5,247,491
CS Share of Total Cost	60%	72%	81%	84%	88%	82%
Total Cost	$369,119	$897,235	$1,344,671	$1,819,655	$1,996,541	$6,427,221

CS = emergency caesarean section delivery procedure; ESMK = Every Second Matters – Ketamine program

For peer review only - http://bmjopen.bmj.com/site/about/guidelines.xhtml

TABLE 4. COST-EFFECTIVENESS RESULTS WITH SENSITIVITY ANALYSIS FOR KEY MODEL UNCERTAINTIES

	Base case impact	Base case + higher CS cost	Base case + higher CS cost and ESMK program Cost	Pessimistic impact	Pessimistic impact + higher CS cost	Pessimistic impact + higher CS cost and ESMK program Cost
**Input Parameters**						
Population rate of Emergency CS	15%	15%	15%	10%	10%	10%
Emergency CS gap attributable to anaesthesia	30%	30%	30%	10%	10%	10%
Incremental CS procedure cost*	$183	$366	$366	$183	$366	$366
Program Cost Multiplier	1x	1x	2x	1x	1x	2x
**Outcomes**						
Undiscounted Program Cost	$1.18m	$1.18m	$2.36m	$1.18m	$1.18m	$2.36m
Undiscounted Total Cost	$6.43m	$11.68m	$12.85m	$1.65m	$2.12m	$3.30m
Additional Emergency CS	28,704	28,704	28,704	2,580	2,580	2,580
Maternal Lives Saved	316	316	316	28	28	28
Fetal Lives Saved	4,736	4,736	4,736	426	426	426
**Incremental Cost-effectiveness Ratio**						
Cost per LY (discounted)	$44	$79	$87	$125	$161	$251
GDPpc per LY (discounted)	2.8%	5.1%	5.6%	8.0%	10.3%	16.1%
Cost per Maternal LY (discounted)	$779	$1,412	$1,557	$2,246	$2,880	$4,492
GDPpc per Maternal LY (discounted)	50%	91%	100%	144%	185%	288%

*excess cost of Caesarean delivery cost above the cost of normal vaginal delivery
CS = caesarean delivery procedure, LY = life-year gained, ESMK = Every Second Matters -Ketamine program

For peer review only - http://bmjopen.bmj.com/site/about/guidelines.xhtml

Figure 1. Schematic Diagram of Decision Analysis Model

Insert Figure 1 Here

For peer review only

For peer review only - http://bmjopen.bmj.com/site/about/guidelines.xhtml

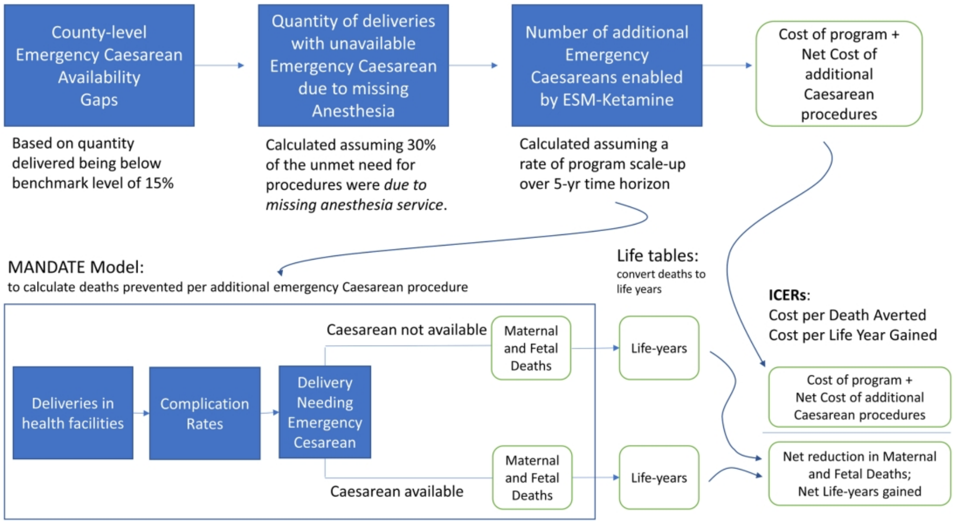

Schematic Diagram of Decision Analytic Model

229x129mm (300 x 300 DPI)

Supplementary Material

Table S1. MANDATE Input Parameters and Resulting Caesarean Procedures

OBSTRUCTED LABOR

FIRST Stage OL Treatment	Baseline	Improved	Difference
Penetration rate in the Hospital	*0.75*	*0.99*	
Utilization rate in the Hospital	*0.6*	*0.99*	
Efficacy (% reduction in condition) in the Hospital	1		
Efficacy (% reduction in Other Fetal Death from OL) in the Hospital	0.95		
Efficacy (% reduction in Ruptured Uterus) in the Hospital	0.95		
Efficacy (% reduction in Birth Asphyxia) in the Hospital	0.85		
Efficacy (% reduction in Maternal Sepsis) in the Hospital	0.85		
Efficacy (% reduction in Neonatal SBI) in the Hospital	0.85		
Efficacy (% reduction in PPH Atonic Uterus) in the Hospital	0.85		
Number CS received	3046	6768	3722

SECOND Stage OL Treatment	Baseline	Improved	Difference
Penetration rate in the Hospital	*0.75*	*0.99*	
Utilization rate in the Hospital	*0.6*	*0.99*	
Efficacy (% reduction in condition) in the Hospital	1		
Efficacy (% reduction in Other Fetal Death from OL) in the Hospital	0.95		
Efficacy (% reduction in Ruptured Uterus) in the Hospital	0.95		
Efficacy (% reduction in Birth Asphyxia) in the Hospital	0.85		
Efficacy (% reduction in Maternal Sepsis) in the Hospital	0.85		
Efficacy (% reduction in Neonatal SBI) in the Hospital	0.85		

For peer review only - http://bmjopen.bmj.com/site/about/guidelines.xhtml

For peer review only

Efficacy (% reduction in PPH Atonic Uterus) in the Hospital	0.85		
Number CS	2543	5652	3109
FIRST Stage Protracted OL Treatment (Late CS - efficacy lower)	Baseline	Improved	Difference
Penetration rate in the Hospital	*0.75*	*0.99*	
Utilization rate in the Hospital	*0.95*	*0.99*	
Efficacy (% reduction in condition) in the Hospital	1		
Efficacy (% reduction in Other Fetal Death from OL) in the Hospital	0.1		
Efficacy (% reduction in Ruptured Uterus) in the Hospital	0.7		
Efficacy (% reduction in Birth Asphyxia) in the Hospital	0.2		
Efficacy (% reduction in Maternal Sepsis) in the Hospital	0.4		
Efficacy (% reduction in Neonatal SBI) in the Hospital	0.2		
Efficacy (% reduction in PPH Atonic Uterus) in the Hospital	0.65		
Number CS	4491	3164	-1327
SECOND Stage Protracted OL Treatment	Baseline	Improved	Difference
Penetration rate in the Hospital	*0.75*	*0.99*	
Utilization rate in the Hospital	*0.95*	*0.99*	
Efficacy (% reduction in condition) in the Hospital	1		
Efficacy (% reduction in Other Fetal Death from OL) in the Hospital	0.1		
Efficacy (% reduction in Ruptured Uterus) in the Hospital	0.7		
Efficacy (% reduction in Birth Asphyxia) in the Hospital	0.2		
Efficacy (% reduction in Maternal Sepsis) in the Hospital	0.4		
Efficacy (% reduction in Neonatal SBI) in the Hospital	0.2		
Efficacy (% reduction in Atonic Uterus) in the Hospital	0.65		
Number CS	3134	2746	-388
	Baseline	Improved	Difference
**TOTAL CS FOR OBSTRUCTED LABOR**	**13214**	**18330**	**5116**

For peer review only - http://bmjopen.bmj.com/site/about/guidelines.xhtml

PRE-ECLAMPSIA AND ECLAMPSIA

Early Low Rate C-section Technology Interventions	Baseline	Improved	Difference
Penetration	*0.75*	*0.99*	*0.24*
Utilization	*0.4*	*0.99*	*0.59*
Mild PE Maternal Efficacy	0.95		
Mild PE Fetal Efficacy	0.5		
Severe PE Maternal Efficacy	0.8		
SeverePE Fetal Efficacy	0.3		
Mild PE Maternal 1st Seizure Efficacy	0.95		
Severe PE Maternal 1st Seizure Efficacy	0.8		
CS Numbers	1047	3489	2442

Early High Rate C-section Technology Interventions (i.e. C-section or induction (to prevent eclampsia if mother is diagnosed with severe preeclampsia)	Baseline	Improved	Difference
Penetration	*0.75*	*0.99*	*0.24*
Utilization	*0.8*	*0.99*	*0.19*
Mild PE Maternal Efficacy	0.6		
Mild PE Fetal Efficacy	0.6		
Severe PE Maternal Efficacy	0.8		-0.8
SeverePE Fetal Efficacy	0.3		
Mild PE Maternal 1st Seizure Efficacy	0.6		
Severe PE Maternal 1st Seizure Efficacy	0.8		
CS Numbers	982	1636	654

Standard C-section Technology Interventions (for those DIAGNOSED WITH ECLAMPSIA that did not have early CS and that have intra and ante seizure)	Baseline	Improved	Difference
Penetration	*0.75*	*0.99*	*0.24*
Utilization	*0.8*	*0.99*	*0.19*
Maternal 1st&Multiple Seizure Efficacy	0.75		
Mild PE Fetal Efficacy	0.4		
Severe PE Maternal 1st&Multiple Seizure Efficacy	0.75		
Severe PE Fetal Efficacy	0.4		
CS Numbers	189	306	117

Standard C-section Technology Interventions (for those DIAGNOSED WITH ECLAMPSIA that did not have early CS and that have intra and ante seizure)	Baseline	Improved	Difference

For peer review only - http://bmjopen.bmj.com/site/about/guidelines.xhtml

Penetration	*0.75*	*0.99*	*0.24*
Utilization	*0.8*	*0.99*	*0.19*
Maternal 1st&Multiple Seizure Efficacy	0.75		
Mild PE Fetal Efficacy	0.4		
Severe PE Maternal 1st&Multiple Seizure Efficacy	0.75		
Severe PE Fetal Efficacy	0.4		
CS Numbers	23	38	15
	Baseline	Improved	Difference
**TOTAL C/S FOR PRE-ECLAMPSIA AND ECLAMPSIA**	**2241**	**5469**	**3228**

ABRUPTION, PREVIA, RUPTURED UTERUS

AIPH due to Significant Abruption Treatment (Early Stage)	Baseline	Improved	Difference
Penetration rate in the Hospital	*0.75*	*0.99*	*0.24*
Utilization rate in the Hospital	*0.2*	*0.99*	*0.79*
Efficacy (% reduction in condition) in the Hospital	0.9		
Efficacy (% reduction in Birth Asphyxia)	0.85		
Efficacy (% reduction in Fetal Deaths)	0.85		
Number CS received	71	475	404
AIPH due to Significant Abruption Treatment (Late Stage)	Baseline	Improved	Difference
Penetration rate in the Hospital	*0.75*	*0.99*	*0.24*
Utilization rate in the Hospital	*0.6*	*0.99*	*0.39*
Efficacy (% reduction in condition) in the Hospital	0.7		
Efficacy (% reduction in Birth Asphyxia)	0.2		
Efficacy (% reduction in Fetal Deaths)	0.1		
Number CS received	424	805	381
Placenta Previa Treatment (Early Stage)	Baseline	Improved	Difference
Penetration rate in the Hospital	*0.75*	*0.99*	*0.24*
Utilization rate in the Hospital	*0.2*	*0.99*	*0.79*
Efficacy (% reduction in condition) in the Hospital	0.9		
Efficacy (% reduction in Birth Asphyxia)	0.85		
Efficacy (% reduction in Fetal Deaths)	0.95		
Number CS received	24	159	135
Placenta Previa Treatment (Late Stage)	Baseline	Improved	Difference
Penetration rate in the Hospital	*0.75*	*0.99*	*0.24*
Utilization rate in the Hospital	*0.6*	*0.99*	*0.39*

For peer review only - http://bmjopen.bmj.com/site/about/guidelines.xhtml

	Baseline	Improved	Difference
Efficacy (% reduction in condition) in the Hospital	0.7		
Efficacy (% reduction in Birth Asphyxia)	0.2		
Efficacy (% reduction in Fetal Deaths)	0.1		
Number CS received	142	263	121
Ruptured Uterus Treatment (Early Stage)	Baseline	Improved	Difference
Penetration rate in the Hospital	*0.75*	*0.99*	*0.24*
Utilization rate in the Hospital	*0.25*	*0.99*	*0.74*
Efficacy (% reduction in condition) in the Hospital	0.75		
Efficacy (% reduction in Birth Asphyxia)	0.85		
Efficacy (% reduction in Fetal Deaths)	0.85		
Number CS received	28	148	120
Ruptured Uterus Treatment (Late Stage)	Baseline	Improved	Difference
Penetration rate in the Hospital	*0.75*	*0.99*	*0.24*
Utilization rate in the Hospital	*0.75*	*0.99*	*0.24*
Efficacy (% reduction in condition) in the Hospital	0.55		
Efficacy (% reduction in Birth Asphyxia)	0.1		
Efficacy (% reduction in Fetal Deaths)	0.1		
Number CS received	203	284	81
	Baseline	Improved	Difference
**TOTAL C/S FOR ABRUPTION, PREVIA, RUPTURED UTERUS**	**892**	**2134**	**1242**

FETAL DISTRESS

SFD- Multiple births during First Stage Labor Treatment	Baseline	Improved	Difference
Penetration rate in the Hospital	*0.75*	*0.99*	*0.24*
Utilization rate in the Hospital	*0.4*	*0.99*	*0.59*
Efficacy (% reduction in condition) in the Hospital	0.9		
Efficacy (% reduction in Birth Asphyxia)	0.5		
Number CS received	52	173	121
SFD- Multiple births during Second Stage Labor Final Treatment	Baseline	Improved	Difference
Penetration rate in the Hospital	*0.75*	*0.99*	*0.24*
Utilization rate in the Hospital	*0.4*	*0.99*	*0.59*
Efficacy (% reduction in condition) in the Hospital	0.9		
Efficacy (% reduction in Birth Asphyxia)	0.5		
Number CS received	21	69	48

For peer review only - http://bmjopen.bmj.com/site/about/guidelines.xhtml

SFD- IUGR during First Stage Labor Treatment	Baseline	Improved	Difference
Penetration rate in the Hospital	*0.75*	*0.99*	*0.24*
Utilization rate in the Hospital	*0.4*	*0.99*	*0.59*
Efficacy (% reduction in condition) in the Hospital	0.9		
Efficacy (% reduction in Birth Asphyxia)	0.5		
Number CS received	499	1663	1164

SFD- IUGR during Second Stage Labor Final Treatment	Baseline	Improved	Difference
Penetration rate in the Hospital	*0.75*	*0.99*	*0.24*
Utilization rate in the Hospital	*0.4*	*0.99*	*0.59*
Efficacy (% reduction in condition) in the Hospital	0.9		
Efficacy (% reduction in Birth Asphyxia)	0.5		
Number CS received	199	664	465

SFD- Breech during First Stage Labor Treatment	Baseline	Improved	Difference
Penetration rate in the Hospital	*0.75*	*0.99*	*0.24*
Utilization rate in the Hospital	*0.4*	*0.99*	*0.59*
Efficacy (% reduction in condition) in the Hospital	0.9		
Efficacy (% reduction in Birth Asphyxia)	0.5		
Number CS received	52	172	120

SFD- Breech during Second Stage Labor Final Treatment	Baseline	Improved	Difference
Penetration rate in the Hospital	*0.75*	*0.99*	*0.24*
Utilization rate in the Hospital	*0.4*	*0.99*	*0.59*
Efficacy (% reduction in condition) in the Hospital	0.9		
Efficacy (% reduction in Birth Asphyxia)	0.5		
Number CS received	21	69	48

SFD- Cord Accident during First Stage Labor Treatment	Baseline	Improved	Difference
Penetration rate in the Hospital	*0.75*	*0.99*	*0.24*
Utilization rate in the Hospital	*0.4*	*0.99*	*0.59*
Efficacy (% reduction in condition) in the Hospital	0.9		
Efficacy (% reduction in Birth Asphyxia)	0.5		
Number CS received	203	678	475

SFD- Cord Accident during Second Stage Labor Final Treatment	Baseline	Improved	Difference

For peer review only - http://bmjopen.bmj.com/site/about/guidelines.xhtml

	Baseline	Improved	Difference
Penetration rate in the Hospital	*0.75*	*0.99*	*0.24*
Utilization rate in the Hospital	*0.4*	*0.99*	*0.59*
Efficacy (% reduction in condition) in the Hospital	0.9		
Efficacy (% reduction in Birth Asphyxia)	0.5		
Number CS received	81	271	190

SFD- Other during First Stage Labor Treatment	Baseline	Improved	Difference
Penetration rate in the Hospital	*0.75*	*0.99*	*0.24*
Utilization rate in the Hospital	*0.4*	*0.99*	*0.59*
Efficacy (% reduction in condition) in the Hospital	0.9		
Efficacy (% reduction in Birth Asphyxia)	0.5		
Number CS received	598	1993	1395

SFD- Other during Second Stage Labor Final Treatment	Baseline	Improved	Difference
Penetration rate in the Hospital	0.75	0.99	*0.24*
Utilization rate in the Hospital	0.4	0.99	*0.59*
Efficacy (% reduction in condition) in the Hospital	0.9		
Efficacy (% reduction in Birth Asphyxia)	0.5		
Number CS received	239	796	557

	Baseline	Improved	Difference
**TOTAL C/S FOR FETAL DISTRESS**	**1965**	**6548**	**4583**
**Total Additional CS All Indications Combined**	**18312**	**32481**	**14169**

For peer review only - http://bmjopen.bmj.com/site/about/guidelines.xhtml

For peer review only

For peer review only - http://bmjopen.bmj.com/site/about/guidelines.xhtml

Table S2. CONSOLIDATED HEALTH ECONOMIC EVALUATION REPORTING STANDARDS (CHEERS) CHECKLIST

CHEERS checklist—Items to include when reporting economic evaluations of health interventions

Section/item	Item No	Recommendation	Reported on Section / Paragraph
**Title and abstract**			
Title	1	Identify the study as an economic evaluation or use more specific terms such as "cost-effectiveness analysis", and describe the interventions compared.	Title Page
Abstract	2	Provide a structured summary of objectives, perspective, setting, methods (including study design and inputs), results (including base case and uncertainty analyses), and conclusions.	Abstract
**Introduction**			
Background and objectives	3	Provide an explicit statement of the broader context for the study.	Introduction, Paragraphs 1-3
		Present the study question and its relevance for health policy or practice decisions.	Introduction, Paragraph 4
**Methods**			
Target population and subgroups	4	Describe characteristics of the base case population and subgroups analysed, including why they were chosen.	Methods, Paragraphs 1,7
Setting and location	5	State relevant aspects of the system(s) in which the decision(s) need(s) to be made.	Methods, Paragraphs 1
Study perspective	6	Describe the perspective of the study and relate this to the costs being evaluated.	Methods, line 108
Comparators	7	Describe the interventions or strategies being compared and state why they were chosen.	Abstract, Introduction line 50, Methods line 78, line 85
Time horizon	8	State the time horizon(s) over which costs and consequences are being evaluated and say why appropriate.	Methods, line 129
Discount rate	9	Report the choice of discount rate(s) used for costs and outcomes and say why appropriate.	Methods, line 131
Choice of health outcomes	10	Describe what outcomes were used as the measure(s) of benefit in the evaluation and their relevance for the type of analysis performed.	Methods, Paragraphs 33-35
Measurement of effectiveness	11a	*Single study-based estimates:* Describe fully the design features of the single	Not applicable

For peer review only - http://bmjopen.bmj.com/site/about/guidelines.xhtml

Section/item	Item No	Recommendation	Reported on Section / Paragraph
		effectiveness study and why the single study was a sufficient source of clinical effectiveness data.	
	11b	*Synthesis-based estimates*: Describe fully the methods used for identification of included studies and synthesis of clinical effectiveness data.	Methods, lines 52-106
Measurement and valuation of preference based outcomes	12	If applicable, describe the population and methods used to elicit preferences for outcomes.	Not applicable
Estimating resources and costs	13a	*Single study-based economic evaluation:* Describe approaches used to estimate resource use associated with the alternative interventions. Describe primary or secondary research methods for valuing each resource item in terms of its unit cost. Describe any adjustments made to approximate to opportunity costs.	Not applicable
	13b	*Model-based economic evaluation:* Describe approaches and data sources used to estimate resource use associated with model health states. Describe primary or secondary research methods for valuing each resource item in terms of its unit cost. Describe any adjustments made to approximate to opportunity costs.	Methods, line 108-127
Currency, price date, and conversion	14	Report the dates of the estimated resource quantities and unit costs. Describe methods for adjusting estimated unit costs to the year of reported costs if necessary. Describe methods for converting costs into a common currency base and the exchange rate.	Methods, line 123-125
Choice of model	15	Describe and give reasons for the specific type of decision-analytical model used. Providing a figure to show model structure is strongly recommended.	Methods, line 52-54, 80-85; Figure 1
Assumptions	16	Describe all structural or other assumptions underpinning the decision-analytical model.	Methods, line 52-166; Figure 1; Tables 1; Suppl material
Analytical methods	17	Describe all analytical methods supporting the evaluation. This could include methods for dealing with skewed, missing, or censored data; extrapolation methods; methods for pooling data; approaches to	Methods, line 52-166, Figure1;

For peer review only - http://bmjopen.bmj.com/site/about/guidelines.xhtml

Section/item	Item No	Recommendation	Reported on Section / Paragraph
		validate or make adjustments (such as half cycle corrections) to a model; and methods for handling population heterogeneity and uncertainty.	
**Results**			
Study parameters	18	Report the values, ranges, references, and, if used, probability distributions for all parameters. Report reasons or sources for distributions used to represent uncertainty where appropriate. Providing a table to show the input values is strongly recommended.	Methods, line 52-166; Tables 1; Figure 1; Table S1, S2, S4, S6 in Supplementary Material
Incremental costs and outcomes	19	For each intervention, report mean values for the main categories of estimated costs and outcomes of interest, as well as mean differences between the comparator groups. If applicable, report incremental cost-effectiveness ratios.	Results, 168-222; Table 4, Table S5
Characterising uncertainty	20a	*Single study-based economic evaluation:* Describe the effects of sampling uncertainty for the estimated incremental cost and incremental effectiveness parameters, together with the impact of methodological assumptions (such as discount rate, study perspective).	Not applicable
	20b	*Model-based economic evaluation:* Describe the effects on the results of uncertainty for all input parameters, and uncertainty related to the structure of the model and assumptions.	Results, line 215-222; Table 4
Characterising heterogeneity	21	If applicable, report differences in costs, outcomes, or cost-effectiveness that can be explained by variations between subgroups of patients with different baseline characteristics or other observed variability in effects that are not reducible by more information.	Not applicable
**Discussion**			
Study findings, limitations, generalisability, and current knowledge	22	Summarise key study findings and describe how they support the conclusions reached. Discuss limitations and the generalisability of the findings and how the findings fit with current knowledge.	Discussion
**Other**			
Source of funding	23	Describe how the study was funded and the role of the funder in the identification, design, conduct, and reporting of the	Funding information page 17

For peer review only - http://bmjopen.bmj.com/site/about/guidelines.xhtml

Section/item	Item No	Recommendation	Reported on Section / Paragraph
		analysis. Describe other non-monetary sources of support.	
Conflicts of interest	24	Describe any potential for conflict of interest of study contributors in accordance with journal policy. In the absence of a journal policy, we recommend authors comply with International Committee of Medical Journal Editors recommendations.	Competing interests page 18

Table adapted from the Consolidated Health Economic Evaluation Reporting Standards (CHEERS) Checklist of items to include when reporting economic evaluations of health interventions.[1]

REFERENCE:

1. Husereau D, Drummond M, Petrou S, Carswell C, Moher D, Greenberg D, et al. Consolidated Health Economic Evaluation Reporting Standards (CHEERS)—Explanation and Elaboration: A Report of the ISPOR Health Economic Evaluation Publication Guidelines Good Reporting Practices Task Force. Value Health. 2013;16: 231–250. doi:10.1016/j.jval.2013.02.002

For peer review only

TABLE S3· COUNTY INDICATORS, 2018

Region	County	Target	Skilled birth attendant present (%)	Population CS rate (%)	Facility CS rate (%)	Operating Rooms	Expected Births	Expected Emergency Caesarean delivery	Current Caesarean delivery
Central	Nyandarua	Yes	85·3	9·0	10·6	8	23,830	3,575	2,145
	Murang'a	Yes	85·5	11·7	13·7	7	29,596	4,439	3,463
	Nyeri		88·1	13·5	15·3	12	23,926	3,589	3,230
	Kirinyaga		92·3	15·3	16·6	2	18,930	2,839	2,896
	Kiambu		92·6	20·1	21·7	14	76,295	11,444	15,335
Coast	Tana River	Yes	32·2	2·4	7·5	2	16,816	2,522	404
	Kilifi	Yes	52·3	4·4	8·4	6	71,010	10,652	3,124
	Kwale	Yes	50·1	5·3	10·6	3	41,030	6,155	2,175
	Taita Taveta	Yes	62·5	8·7	13·9	1	10,858	1,629	945
	Lamu		47·3	7·3	15·4	3	5,381	807	393
	Mombasa		82·8	13·3	16·1	18	49,198	7,380	6,543
Eastern	Marsabit	Yes	25·8	2·3	8·9	2	8,744	1,312	201
	Kitui	Yes	46·2	5·4	11·7	5	42,087	6,313	2,273
	Isiolo		43·8	6·9	15·8	2	8,168	1,225	564
	Machakos		63·4	11·4	18·0	7	47,372	7,106	5,400
	Makueni		54·6	9·9	18·1	4	34,112	5,117	3,377
	Tharaka-Nithi		76·6	14·5	18·9	4	13,549	2,032	1,965
	Embu		81·5	15·9	19·5	6	19,314	2,897	3,071
	Meru		82·8	18·5	22·3	9	49,678	7,452	9,190
N· Eastern	Mandera	Yes	38·7	1·8	4·7	3	14,894	2,234	268
	Wajir	Yes	21·7	1·3	6·0	4	24,791	3,719	322

For peer review only

	Garissa	Yes	39·8	5·5	13·8	21	22,773	3,416	1,253
Nyanza	Homa Bay	Yes	60·4	3·1	5·1	5	63,227	9,484	1,960
	Siaya	Yes	70·4	4·3	6·1	4	37,571	5,636	1,616
	Nyamira	Yes	74·1	5·2	7·0	1	18,737	2,811	974
	Kisii	Yes	72·8	5·3	7·3	13	46,315	6,947	2,455
	Kisumu	Yes	69·2	6·8	9·8	14	48,045	7,207	3,267
	Migori	Yes	53·4	6·5	12·2	5	54,290	8,144	3,529
Rift Valley	Turkana	Yes	22·8	0·5	2·2	4	33,343	5,001	167
	Elgeyo Marakwet	Yes	65·0	3·5	5·4	6	16,143	2,421	565
	Nandi	Yes	46·8	2·8	6·0	4	38,628	5,794	1,082
	Uasin Gishu	Yes	59·0	4·8	8·1	10	46,411	6,962	2,228
	West Pokot	Yes	27·0	2·2	8·1	2	29,019	4,353	638
	Bomet	Yes	52·2	4·8	9·2	8	48,237	7,236	2,315
	Baringo	Yes	53·8	5·2	9·7	4	22,869	3,430	1,189
	Nakuru	Yes	69·5	6·9	9·9	11	87,345	13,102	6,027
	Samburu	Yes	29·0	2·9	10·0	3	11,242	1,686	326
	Narok	Yes	40·3	5·1	12·7	5	61,305	9,196	3,127
	Trans-Nzoia	Yes	41·8	5·3	12·7	3	50,735	7,610	2,689
	Laikipia	Yes	49·5	6·5	13·1	3	20,083	3,012	1,305
	Kericho		64·4	10·3	16·0	9	35,841	5,376	3,692
	Kajiado		63·2	11·1	17·6	8	44,297	6,645	4,917
Western	Kakamega	Yes	48·6	3·1	6·4	6	71,779	10,767	2,225
	Busia	Yes	58·5	4·0	6·8	7	39,301	5,895	1,572
	Bungoma	Yes	41·4	5·0	12·1	7	83,598	12,540	4,180
	Vihiga	Yes	50·3	6·6	13·1	4	22,004	3,301	1,452
Nairobi	Nairobi		89·1	20·7	23·2	51	197,079	29,562	40,795
**KENYA**		**57**	**61·8**	**8·7**	**14·0**	**340**	**1,879,796**	**281,969**	**162,828**
Target Counties		*33*	*52·9*	*4·9*	*9·3*	*191*	*1,256,657*	*188,498*	*61,459*

CS = caesarean delivery procedure

For peer review only - http://bmjopen.bmj.com/site/about/guidelines.xhtml

TABLE S4· ESM-KETAMINE PROGRAM SCALE UP TARGETS

**Year**	**1**	**2**	**3**	**4**	**5**
ESMK providers trained initially	72	144	144	144	69
ESMK provider trained to offset turnover	5	15	25	35	40
Total ESMK providers in service	72	216	360	504	**573**
Absolute number of QA/QC staff	1	2	2	3	4
Facilities "on-line"	24	72	120	168	**191**
Coverage	13%	38%	63%	88%	100%

ESMK= Every Second Matters -Ketamine program, QA/QC = Quality Assurance/ Quality Control

TABLE S5· ESM-KETAMINE PROGRAM COST PROJECTION· BASE CASE, UNDISCOUNTED

Year	1	2	3	4	5	Total
Training (initial)	$61,200	$122,400	$122,400	$122,400	$58,650	$487,050
Training (replacement)	$4,250	$12,750	$21,250	$29,750	$34,000	$102,000
QA/QC	$16,400	$32,800	$32,800	$49,200	$65,600	$196,800
Coordination	$48,000	$48,000	$48,000	$48,000	$48,000	$240,000
Overhead	$19,478	$32,393	$33,668	$37,403	$30,938	$153,880
Total	$149,328	$248,343	$258,118	$286,753	$237,188	**$1,179,730**

QA/QC = Quality Assurance/Quality Control

For peer review only - http://bmjopen.bmj.com/site/about/guidelines.xhtml

FIG S1. ANNUAL COST OF SCALING UP ESM-KETAMINE PROGRAM, BY ACTIVITY

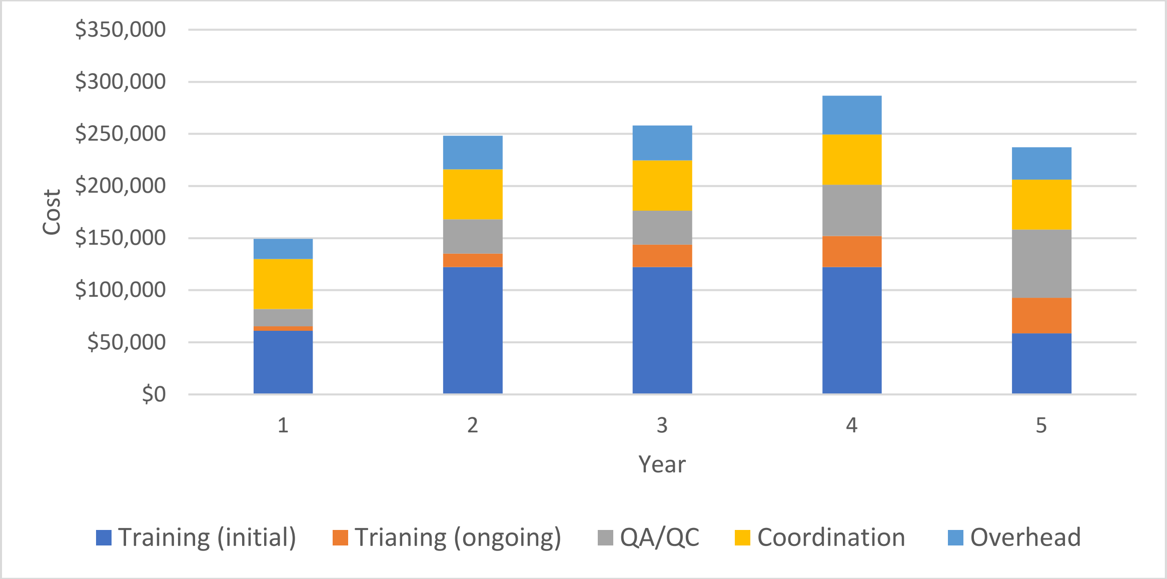

QA/QC = Quality Assurance/Quality Control

For peer review only - http://bmjopen.bmj.com/site/about/guidelines.xhtml

TABLE S6. EXPECTED HEALTH IMPACT OF EMERGENCY CAESAREAN DELIVERY, WEIGHTED AVERAGE LIVES SAVED ACROSS ALL INDICATIONS.

	**Lives Saved**	**Lives saved per Caesarean delivery**	**Number of procedures to save 1 life**
Maternal	154	0·011	92
Foetal	2,339	0·165	6·1
Total	2,493	0·178	5·7

For peer review only

TABLE S7. FOETAL AND MATERNAL DEATHS IN 2017 ATTRIBUTABLE TO COMPLICATIONS THAT INDICATE EMERGENCY CAESAREAN DELIVERY UNDER A BASELINE SCENARIO AND AN "IMPROVED" SCENARIO IN WHICH THERE ARE NO BOTTLENECKS TO EMERGENCY CAESAREAN DELIVERY IN HOSPITAL FACILITIES.

**Complication**	**Foetal Deaths: Baseline**	**Foetal Deaths: Improved**	**Foetal Deaths Prevented**	**Percent of Foetal Deaths Prevented**
Foetal Abruption	5,093	4982	111	5%
Foetal Previa	508	494	14	1%
Foetal Ruptured Uterus	1,618	1554	64	3%
Foetal PE/E	6,077	5712	365	16%
SFD MB	488	461	28	1%
SFD IUGR	4,899	4635	264	11%
SFD Breech	498	470	27	1%
SFD Cord	1,996	1889	108	5%
SFD De Novo	5,891	5575	316	14%
Foetal OL	7,195	6665	530	23%
NN Sepsis Term	6,490	6395	95	4%
Term BA	12,318	11900	418	18%
Total Foetal Deaths	53,071	50732	2,339	100%

**Complication**	**Maternal Deaths: Baseline**	**Maternal Deaths: Improved**	**Maternal Deaths Prevented**	**Percent of Maternal Deaths Prevented**
Maternal Sepsis	731	717	14	9%
Abruption	348	337	12	8%
Previa	104	100	4	3%
Ruptured Uterus	216	205	11	7%
Atonic Uterus	2,262	2,254	8	5%
PE/E	931	825	106	69%
Total Maternal Deaths	4,592	4,439	154	100%
**Total Deaths**	**57,663**	**55,171**	**2,493**	

For peer review only - http://bmjopen.bmj.com/site/about/guidelines.xhtml

For peer review only

For peer review only - http://bmjopen.bmj.com/site/about/guidelines.xhtml
